# Flexibility *In Vitro* of Amino Acid 226 in the Receptor-Binding Site of an H9 Subtype Influenza A Virus and Its Effect *In Vivo* on Virus Replication, Tropism, and Transmission

**DOI:** 10.1128/JVI.02011-18

**Published:** 2019-03-05

**Authors:** Adebimpe O. Obadan, Jefferson Santos, Lucas Ferreri, Andrew J. Thompson, Silvia Carnaccini, Ginger Geiger, Ana S. Gonzalez Reiche, Daniela S. Rajão, James C. Paulson, Daniel R. Perez

**Affiliations:** a Poultry Diagnostic and Research Center, Department of Population Health, University of Georgia, Athens, Georgia, USA; b Department of Molecular Medicine, and Immunology & Microbiology, The Scripps Research Institute, La Jolla, California, USA; c Department of Genetics and Genomic Sciences, Icahn School of Medicine at Mount Sinai, New York, New York, USA; St. Jude Children's Research Hospital

**Keywords:** H9 subtype, avian viruses, evolution, quail, receptor, sialic acid, transmission, zoonosis

## Abstract

Influenza A viruses (IAVs) remain a significant public health threat, causing more than 300,000 hospitalizations in the United States during the 2015–2016 season alone. While only a few IAVs of avian origin have been associated with human infections, the ability of these viruses to cause zoonotic infections further increases the public health risk of influenza. Of these, H9N2 viruses in Asia are of particular importance as they have contributed internal gene segments to other emerging zoonotic IAVs. Notably, recent H9N2 viruses have acquired molecular markers that allow for a transition from avian-like to human-like terminal sialic acid (SA) receptor recognition via a single amino acid change at position 226 (H3 numbering), from glutamine (Q226) to leucine (L226), within the hemagglutinin (HA) receptor-binding site (RBS). We sought to determine the plasticity of amino acid 226 and the biological effects of alternative amino acids on variant viruses. We created a library of viruses with the potential of having any of the 20 amino acids at position 226 on a prototypic H9 HA subtype IAV. We isolated H9 viruses that carried naturally occurring amino acids, variants found in other subtypes, and variants not found in any subtype at position 226. Fitness studies in quails revealed that some natural amino acids conferred an *in vivo* replication advantage. This study shows the flexibility of position 226 of the HA of H9 influenza viruses and the resulting effect of single amino acid changes on the phenotype of variants *in vivo* and *in vitro*.

**IMPORTANCE** A single amino acid change at position 226 in the hemagglutinin (HA) from glutamine (Q) to leucine (L) has been shown to play a key role in receptor specificity switching in various influenza virus HA subtypes, including H9. We tested the flexibility of amino acid usage and determined the effects of such changes. The results reveal that amino acids other than L226 and Q226 are well tolerated and that some amino acids allow for the recognition of both avian and human influenza virus receptors in the absence of other changes. Our results can inform better avian influenza virus surveillance efforts as well as contribute to rational vaccine design and improve structural molecular dynamics algorithms.

## Abstract (precis)

A single amino acid change at position 226 in the hemagglutinin (HA) from glutamine (Q) to leucine (L) has been shown to play a key role in receptor specificity switching in various influenza virus HA subtypes, including H9. We tested the flexibility of amino acid usage and determined the effects of such changes. The results reveal that amino acids other than L226 and Q226 are well tolerated and that some amino acids allow for the recognition of both avian and human influenza virus receptors in the absence of other changes. Our results can inform better avian influenza virus surveillance efforts as well as contribute to rational vaccine design and improve structural molecular dynamics algorithms.

## INTRODUCTION

The hemagglutinin (HA) of influenza A viruses (IAVs) plays a central role in the viral life cycle through its involvement in receptor recognition, virus attachment, membrane fusion, and virus entry (1). The HA is also the most significant target of neutralizing antibodies during infection. Antigenic differences led to the classification of the HA into 18 subtypes (H1 to H18) that are phylogenetically separated into two major groups: group 1 contains H1, H2, H5, H6, H8, H9, H11, H12, H13, H16, H17, and H18, and group 2 contains H3, H4, H7, H10, H14, and H15. The HA is a single-pass type I transmembrane glycoprotein present as a homotrimer on the virus surface that extends ∼130 Å from the membrane. Each HA monomer contains two subunits, HA1 and HA2, produced by protease cleavage of the inactive precursor, HA0. The globular head of HA1 carries a shallow grove, the receptor-binding site (RBS), which is responsible for the receptor recognition function (2–4). On the target cell, the RBS typically binds glycan structures terminating in *N*-acetylneuraminic acid (also referred to as sialic acid [SA]), although horse-adapted IAV strains develop a preference for *N*-glycolylneuraminic acid (5). In nature, SAs are linked to the penultimate galactose (Gal) in two major conformations: α2-3SA or α2-6SA. The structure of the RBS is conserved across all HA subtypes and is made up of the 130 loop (residues 135 to 138), 190 helix (residues 190 to 198), and 220 loop (residues 221 to 228) (6). It is well established that amino acid changes in the RBS, as well as differences in the type of SA linkage expressed in different host species, are major determinants of the host range and tissue tropism of IAVs (7–9). Through hydrogen-bonding and hydrophobic interactions, the conserved residues tyrosine 98 (Y98), serine 136 (S136), tryptophan 153 (W153), and histidine 183 (H183) (H3 numbering) form the base of the RBS (10). Seminal work by Rogers and Paulson identified that a change at amino acid position 226 from glutamine (Q226) to leucine (L226) within the RBS of the H3 HA subtype altered the SA receptor recognition from an avian-like (α2-3SA) to human-like (α2-6SA) specificity (9). This Q226L mutation, along with the G228S mutation, played an important role in the emergence of the 1968 Hong Kong H3N2 pandemic influenza virus. In general, the HAs of avian-origin IAVs have the Q226 residue, allowing for the recognition of and attachment to α2-3SAs (11, 12). The α2-3SAs are the predominant forms in the digestive tract of wild waterfowl species that are considered the natural hosts of IAVs, where these viruses establish an infection in the intestinal tract and spread by the fecal-oral route through the water (13). In contrast, the HAs of H3 subtype IAVs established in the human population carry L226 at this position (which has transitioned to isoleucine [I226] in all isolates since the early 2000s), allowing binding to α2-6SAs, found primarily in the respiratory tract of humans (14, 15). The Q226L change and its influence on SA recognition are not limited to H3 viruses. This phenomenon has been observed with other subtypes, such as H2, H4, H5, H7, and H9 (16–18).

H9N2 viruses are endemic in most of Asia, the Middle East, and parts of Africa, where they have caused disease outbreaks in chickens, quail, and other minor poultry species (17). H9N2 viruses have been involved in the emergence of zoonotic strains by the contribution of gene segments to, most notably, the goose/Guangdong H5N1 lineage and the Asian-lineage H7N9 and H10N8 viruses, all of which have caused human fatalities (18–22). The expanding geographical spread and enzootic nature of H9N2 viruses as well as the presence of molecular markers favoring transmission to humans and pigs make these viruses of pandemic concern. Older avian H9N2 virus isolates have the Q226 residue (residue 216 in the H9 HA sequence), which provides binding to avian-like receptors, while the vast majority of recent H9N2 strains carry L226, which favors human-like receptor binding (23, 24). It is therefore not surprising that H9N2 viruses are capable of crossing the avian-mammalian host barrier and causing infections in humans (25–28). While the effect of the Q226L change has been well characterized, the plasticity at this position and the resulting effect on receptor recognition and host adaptation are less understood. We sought to determine the flexibility of position 226 in the context of a prototypic H9 virus of the G1 lineage by varying the amino acid at this position using site-directed mutagenesis (29). Our study reveals that position 226 is plastic and can accommodate several different amino acids. *In vivo*, however, strong selection toward L226 was observed in quail, highlighting the limitations of *in vitro* systems to fully recapitulate virus-host interactions in natural hosts. The results provide new insights into the biology of H9N2 influenza viruses and potential avenues for development of live attenuated virus vaccines against the H9 and other influenza virus subtypes.

## RESULTS

### Position 226 of H9 HA is flexible.

**FIG 1:**
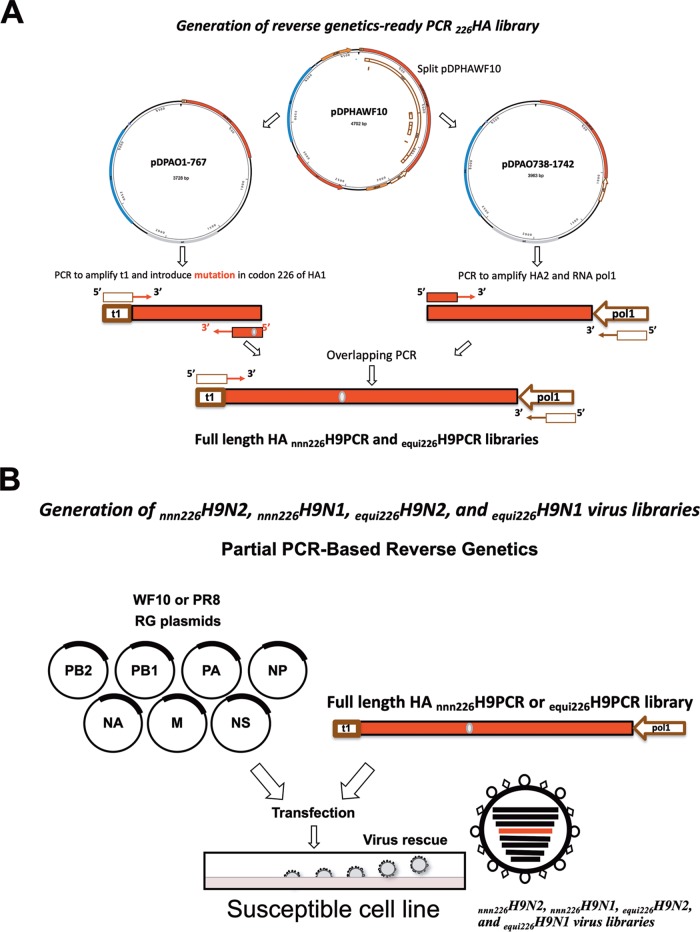
Schematic overview of the steps to generate the degenerate H9 HA PCR product and rescue the H9 HA virus library. (A) The wild-type WF10 HA plasmid was split into 2 plasmids with designed primers (pDPAO1-767 and pDPAO738-1742). The HA PCR product (1 to 767) carrying the mouse RNA polymerase terminator sequence and the degenerate NNN codon at position 226 was generated from the pDPAO1-767 plasmid either with a specific primer with the NNN codon (nnn226) or with a mix of primers able to introduce all 20 amino acids (equi226). Another PCR product with the remaining HA (residues 738 to 1742) and human polymerase 1 promoter was generated from pDPAO738-1742. Using overlapping PCR, a full-length HA was obtained with the degenerate codon at position 226 flanked by the mouse RNA polymerase terminator sequence and the human pol 1 promoter. (B) Generation of virus library by PCR-based reverse genetics using the _226_HA PCR product and 7 plasmids carrying proteins from WF10 (H9N2) or PR8 (H1N1).

To determine the plasticity of amino acids at position 226, two reverse genetics-ready PCR libraries of the H9 HA gene segment were generated: the first one contained a degenerate codon at position 226, and the second one was produced with an equimolar mixture of 20 primers capable of introducing a codon for every possible amino acid at this position (Fig. 1A). The PCR libraries were subsequently used to generate two virus libraries paired with the N2 neuraminidase (NA) gene segment in the background of the A/guinea fowl/Hong Kong/WF10/1999 (H9N2) (WF10) virus (the _nnn226_H9N2 and _equi226_H9N2 virus libraries) (Fig. 1B). We also generated two additional virus libraries paired with the N1 NA gene segment in the background of the laboratory-adapted strain A/Puerto Rico/8/1934 (H1N1) (PR8) (the _nnn226_H9N1 and _equi226_H9N1 virus libraries) (Fig. 1B). The two backgrounds were chosen in order to determine potential biases depending on the HA/NA combination and/or internal gene constellation.

**FIG 2:**
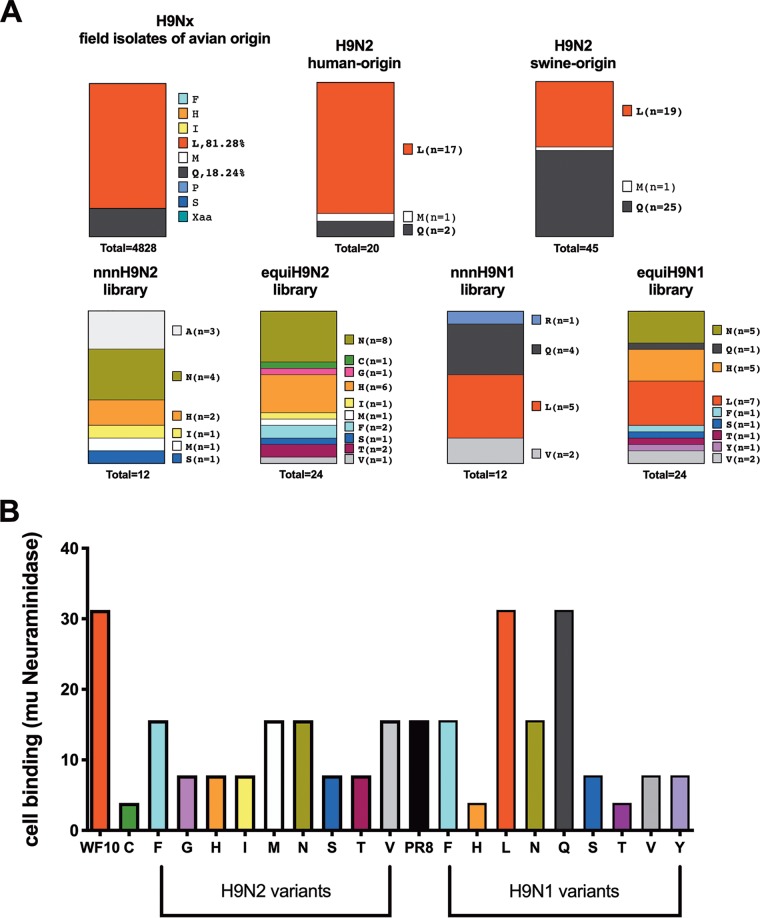
Amino acid diversity at position 226 and impact of mutations on receptor avidity. (A) The amino acid present at position 226 in natural isolates of H9N*x* viruses by species of origin (avian, human, and swine) compared to the amino acid identified experimentally using either the _nnn_226 approach or the _equi_226 approach on an H9N1 or an H9N2 backbone. (B) Receptor-binding avidity of viruses using chicken red blood cells treated with increasing concentrations of neuraminidase from Clostridium perfringens and comparison to that of wild-type WF10 (L226) virus and wild-type PR8 virus.

Following limiting dilution of all four virus libraries in Madin-Darby canine kidney (MDCK) cells, virus clones were analyzed by partial sequencing of the HA gene segment to identify the codon encoding amino acid 226. For simplicity, we refer to variant amino acids (or viruses) as those that carry an amino acid different from L226. Throughout the article we make the distinction of whether a variant amino acid has been found in natural isolates or not. Analysis of 12 individual virus clones from the _nnn226_H9N2 library revealed asparagine (N226, *n* = 4) and alanine (A226, *n* = 3), which have not yet been found in natural isolates, to be the most abundant. Other naturally occurring amino acids included histidine (H226, *n* = 2), isoleucine (I226, *n* = 1), serine (S226, *n* = 1), and methionine (M226, *n* = 1) (Fig. 2A). Interestingly, none of the clones generated using this approach contained the most common amino acids found in nature, either L226 or Q226.

Given that the codon usage for amino acids differs and the probability that the presence of one amino acid in a virus variant may stem from increased codon usage of that amino acid (6 codons code for leucine and serine, whereas 1 codon codes for methionine), we proceeded with an alternative approach to give each amino acid the same representation in the library. Analysis of 24 individual virus clones produced from the _equi226_H9N2 virus library revealed that N226 (*n* = 8) was the most commonly selected amino acid, as was seen with clones obtained from the _nnn226_H9N2 virus library (Fig. 2A). Also consistent with results obtained using the _nnn226_H9N2 virus library, clones produced from the _equi226_H9N2 virus library contained H226 (*n* = 6), I226 (*n* = 1), M226 (*n* = 1), and S226 (*n* = 1). In addition, clone selection from the _equi226_H9N2 virus library resulted in viruses with threonine (T226, *n* = 2), phenylalanine (F226, *n* = 2), cysteine (C226, *n* = 1), glycine (G226, *n* = 1), and valine (V226, *n* = 1). Once again, L226 and Q226 viruses were absent after clonal selection of the _equi226_H9N2 virus library. The absence of these two amino acids is not due to a misrepresentation of the corresponding codons in the preparation of the virus library (as ascertained by next-generation sequencing [NGS] analysis) or a defect in virus rescue. Both of these viruses were independently recovered in the H9N2 background using PCR reverse genetics and the corresponding primer set, indicating that they are viable, as expected (17). Interestingly, considering the H9N2 and H9N1 backgrounds, the yet-to-be-found variant N226 (*n* = 17) and the naturally occurring but rare variant H226 (*n* = 13) were the most frequently obtained among the virus clones. Noteworthy, the naturally occurring L226 (*n* = 12) was the most favored amino acid in HA when paired with the N1 NA (Fig. 2A) but not with the N2 NA, suggesting the presence of a bias in the rescue approach depending on the NA subtype/virus backbone.

### Receptor-binding activity is decreased for most variant viruses.

To investigate receptor-binding activity, we carried out HA assays using chicken red blood cells (RBCs) treated with various concentrations of neuraminidase. As shown in Fig. 2B, all variant viruses had lower receptor function than the WF10 virus. Of note, the viruses with the 2 most commonly found amino acids at position 226, i.e., Q and L, had a receptor-binding capacity similar to that of the WF10 virus. Though the binding for most variants was reduced compared to that of the WF10 virus, the binding for many was similar to that of the control PR8 virus, with the exception of the C226 (H9N2), H226 (H9N1), and T226 (H9N1) viruses, which had a more severely compromised receptor-binding function (Fig. 2B).

### Limited effects in hemagglutination activity by amino acid 226.

Except where noted, we characterized virus clones obtained from the _equi226_H9N2 and _equi226_H9N1 virus libraries. We assessed the ability of the virus clones to agglutinate red blood cells from different species using stocks grown in MDCK cells, as previously described (30, 31). Turkey and chicken red blood cells (RBCs) have both α2-3SAs and α2-6SAs, while horse RBCs have predominantly α2-3SAs. In general, the HA titers of variant viruses were similar or within 1 log_2_ for all viruses tested (Table 1). Differences in agglutinating ability, however, were observed with the horse RBCs. While most H9N2 and H9N1 viruses and the WF10 (L226) virus did not agglutinate horse RBCs, the H9N2 variants with A226, N226, and Q226 and the H9N1 variants with R226 and Y226 agglutinated horse RBCs to various degrees, suggesting that these viruses have the ability to recognize alternative glycan structures.

**TABLE 1:** Hemagglutination and hemagglutination inhibition assay titers of variant viruses^*a*^

Virus	Hemagglutination assay titer			Hemagglutination inhibition assay titer	
	0.5% cRBC	0.5% tRBC	1% hRBC	WF10 antiserum	M226 antiserum
I226 (H9N2)	512	512	0	2,560	2,560
S226 (H9N2)	256	128	0	2,560	2,560
T226 (H9N2)	512	256	0	5,120	2,560
M226 (H9N2)	512	512	0	2,560	2,560
H226 (H9N2)	256	512	0	2,560	2,560
N226 (H9N2)	1,024	512	4	2,560	2,560
F226 (H9N2)	256	256	0	2,560	5,120
V226 (H9N2)	256	256	0	2,560	2,560
C226 (H9N2)	256	128	0	2,560	2,560
G226 (H9N2)	512	256	0	2,560	2,560
L226 (H9N2)	256	256	0	2,560	2,560
G226 (H9N2)	512	512	128	320	640
Y226 (H9N1)	128	512	8	5,120	10,240
R226 (H9N1)	512	512	128	ND	ND
A226 (H9N2)	256	256	64	ND	ND
PR8 (H1N1)	1,024	1,024	0	<10	<10

a cRBC, chicken red blood cells; tRBC, turkey red blood cells; hRBC, horse red blood cells; ND, not done.

### Position 226 does not impact the antigenicity of the H9 HA protein.

Amino acid changes around the receptor-binding site are known to contribute to the antigenic drift of the resulting viruses (32, 33). The L226Q mutation has previously been identified in H9N2 monoclonal antibody escape mutants (34). To evaluate if other amino acids at position 226 can alter the antigenicity of viruses, we carried out hemagglutination inhibition (HI) assays using quail antisera against the WF10 virus and the M226 virus clone (M226 was the only amino acid present in both avian and mammalian hosts, other than L and Q) (Table 1). For all viruses tested, the HI titers were the same or within 1 log_2_ of the homologous HI titer, except with the Q226 virus, which had a titer 3 log_2_ and 2 log_2_ less than the titers obtained for the WF10 and M226 viruses, respectively. Interestingly, the Y226 H9N1 virus showed HI titers better than those observed against the homologous viruses (WF10 and M226), most likely due to its low RBS binding activity.

### Variant viruses retain replicative fitness in cells of mammalian and avian origin.

**FIG 3:**
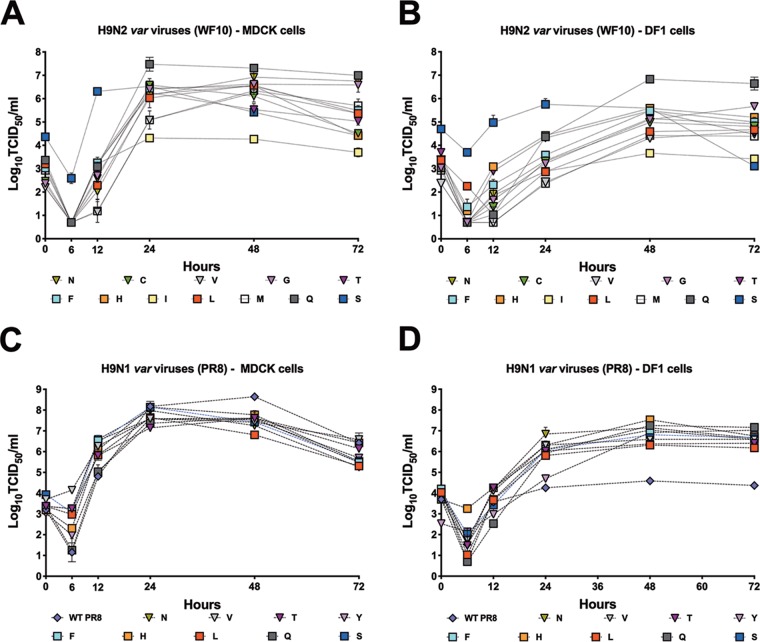
*In vitro* replication of H9N2 and H9N1 variants. Replication of H9N2 and H9N1 viruses in mammalian MDCK cells (A, C) and avian-origin DF1 cells (B, D) at 37°C. Naturally occurring amino acids previously found in H9 HA are shown as squares; others are shown as triangles. Confluent monolayers of MDCK or DF1 cells were infected with viruses at an MOI of 0.01, and supernatant was collected at 0, 6, 12, 24, 48, and 72 hpi. The virus in supernatant collected from MDCK cells was quantified by determination of the TCID_50_ using the Reed and Muench method (62). The plotted data represent means ± standard errors. WT, wild type.

To investigate the contribution of the various amino acids to viral replication, we evaluated and compared the growth kinetics of variant viruses to those of the wild-type WF10 and PR8 viruses in both mammalian and avian cell lines. Confluent canine-origin MDCK cells or chicken-origin DF1 cells were infected at a multiplicity of infection (MOI) of 0.01, and supernatants were collected at different times postinfection. Variant viruses exhibited diverse replication profiles in MDCK and DF1 cells compared to the L226 virus (Fig. 3). At 24 h after infection of MDCK cells, all H9N2 variant viruses replicated to titers similar to those for the L226 virus, with the exception of the I226 (*P* < 0.0001), M226 (*P* < 0.05), and V226 (*P* < 0.05) viruses, which that grew to significantly lower titers, while the Q226 virus (*P* < 0.0001) grew to higher titers than the L226 virus (Fig. 3A). At 72 h postinfection (hpi), the G226 and Q226 viruses replicated to higher titers than the L226 virus (*P* < 0.001 and *P* < 0.0001, respectively). The I226 virus replicated at consistently lower titers during the course of the experiments (*P* < 0.0001) both at 48 and at 72 hpi. In DF1 cells, the pattern of growth of H9N2 variant viruses was similar to the growth kinetics in MDCK cells, the I226 virus replicated to lower titers at every time point except at 24 hpi, and the Q226 virus replicated to higher titers than the other variants tested (Fig. 3B). By 72 hpi, the Q226 and G226 viruses replicated better than the L226 virus, as observed in MDCK cells (*P* < 0.0001 and *P* < 0.01, respectively). The S226 variant virus grew to titers similar to those of the I226 virus at 72 hpi, while there was no difference between the L226 virus and the variant viruses at 72 hpi. In MDCK cells and on the PR8 backbone, all H9N1 viruses replicated to lower titers than wild-type PR8 (H1N1) virus (Fig. 3C). At 48 hpi and by 72 hpi, there were no significant differences between the H9N1 viruses and PR8 virus, except for the L226 (*P* < 0.0001), N226 (*P* < 0.001), and F226, S226, and H226 (*P* < 0.05) viruses, which grew to lower titers in MDCK cells. Interestingly, in DF1 cells, all H9N1 viruses replicated to similar titers and to titers significantly higher (∼2 log_10_) than the titer of the PR8 virus, suggesting that the H9 HA was responsible for improving virus replication in chicken cells (Fig. 3D). Overall, there were no significant adverse effects conferred by the various amino acid changes at position 226 to affect viral replication in mammalian or avian cells.

### Sialic acid receptor specificity is modulated by amino acid 226.

**FIG 4:**
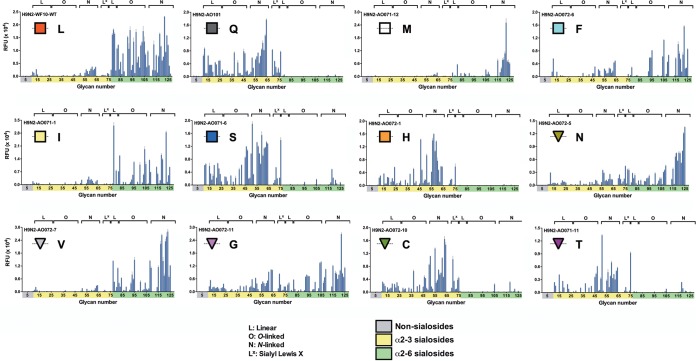
The sialic acid specificity of viruses is dependent on the amino acid at position 226 in glycan array assays. The receptor-binding specificity of a subset of mutant viruses in the H9N2 backbone was determined using a glycan array. L, Q, M, F, I, S, H, N, V, C, T, and G correspond to amino acid mutations at position 226 in the HA of H9N2 viruses. Glycans on the array comprise nonsialoside controls (glycans 1 to 10; gray), α2-3 sialosides (glycans 11 to 79; yellow), and α2-6 sialosides (glycans 80 to 135; green). Glycans are grouped by structure type: L, linear; O, O linked; N, N linked; and L^x^, sialyl-Lewis X. RFU, relative fluorescent units. Plotted data represent means ± standard errors (SEM).

**FIG 5:**
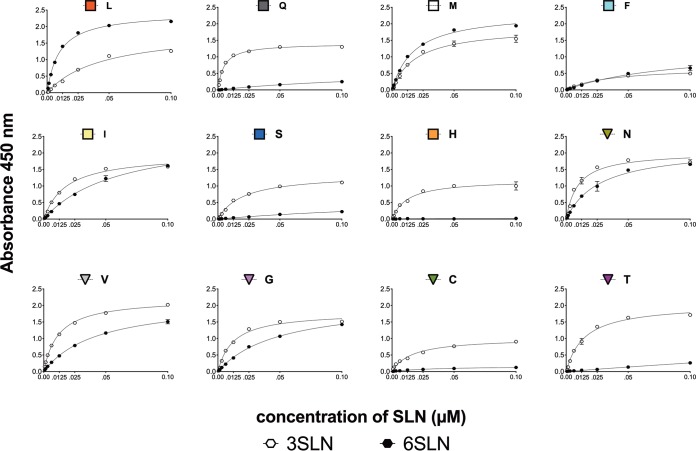
The sialic acid specificity of viruses is dependent on the amino acid at position 226 in solid-phase binding assays. The receptor-binding specificity of mutant viruses on the H9N2 backbone was determined using various concentrations of sialic acid conjugated to biotinylated sialylglycopolymers (3SLN and 6SLN) via direct solid-phase binding assays. The *y* axis represents absorbance (optical density) values at 450 nm. The *x* axis corresponds to the concentrations of serially diluted 3SLN or 6SLN sialylglycopolymers. As expected, the H9N2 L226 virus bound mainly 6SLN-PAA-biotin while the Q226, C226, H226, S226, and T226 viruses bound more to the 3SLN-PAA-biotin moiety. The F226, G226, and I226 viruses showed a preference for both 3SLN and 6SLN sugars. The figure represents the results of a prototypical assay with samples run in duplicate.

We analyzed α2-3SA and α2-6SA receptor specificity using two independent assays: we performed glycan binding assays using a custom glycan microarray with linear, O-linked, and N-linked sialosides (Fig. 4) and solid-phase direct binding assays using synthetic high-molecular-weight biotinylated polyacrylamide probes having multiple copies of monospecific sialyloligosaccharide moieties (3SLN and 6SLN, respectively; Fig. 5) (35). The findings on the glycan arrays and solid-phase assays were, in general, consistent. The L226 and Q226 variants exhibited preferences identical to those reported previously (17). The L226 virus bound almost all α2-6SA structures on the array, resulting in fewer gaps and a less comb-like appearance. The Q226, C226, H226, S226, and T226 viruses bound exclusively to α2-3SA on the glycan array, also corroborated by the solid-phase binding data. We did observe limited binding of these variants to some α2-6 N-linked sialosides with 4 to 5 LacNac repeats, with the exception of the H226 virus, where binding was limited to only the α2-3SAs. Also, while the Q226, C226, and S226 variants bound the sialyl-Lewis X receptors on the array, this was more limited with the H226 and T226 variants. The preference for the M226 variant was restricted mainly to the N-linked subset of the α2-6SA-terminating sialosides. This was in contrast to the solid-phase data, where M226 bound both the 3SLN and 6SLN sugars equally well. The I226 and V226 viruses bound to α2-6SAs in linear as well as O- and N-linked conformations, but with different binding patterns. A more expanded binding specificity phenotype was seen with the F226, G226, and N226 viruses. These 3 variants bound fairly consistently to linear and N-linked α2-3SA, with variable and lower binding to O-linked α2-3SA. Further inspection showed that the F226 virus bound fewer linear α2-3SAs and had no interaction with the Lewis X α2-3SAs, unlike the G226 and N226 viruses. In the α2-6SA configuration, the F226 virus did not bind linear sugars and showed a preference for longer branched receptors. The binding phenotypes for both the N226 and G226 viruses were similar in appearance. Among all viruses analyzed and considering all binding assays, the L226 variant showed the best overall α2-6SA binding preference. In contrast, the Q226 variant showed the best overall α2-3SA binding activity. The rest of the viruses showed less overall avidity, with either dual or α2-3SA binding preferences.

### The intensity and pattern of virus attachment differ with the amino acid at position 226 in quail trachea.

**FIG 6:**
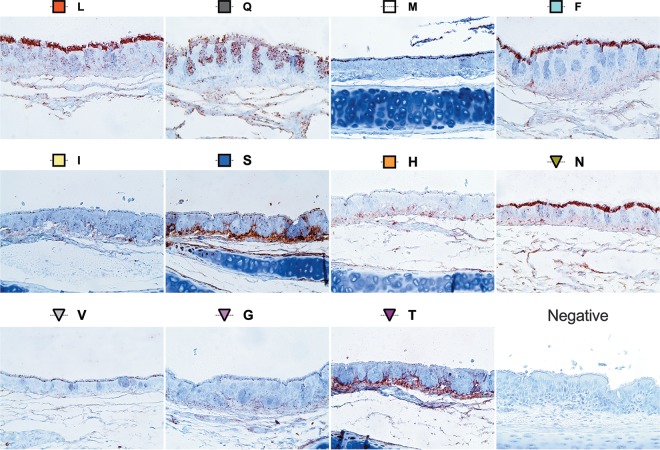
Pattern of virus attachment to quail tracheal tissues. The attachment patterns of the *var* viruses to quail tracheal tissue sections were determined. Virus attachment was seen as red staining on the tracheal surface epithelium. The intensity of binding to the surface epithelium of the trachea was scored as none (−), low (+), moderate (++), and intense (+++) (Table 2). Representative bright-field images are shown.

The pattern of influenza virus attachment (PVA) in the respiratory tract is an indicator of virus tropism, which can determine the efficiency of viral replication and transmission (72). Since the respiratory tract and not the intestinal tract is the major site of replication of avian influenza viruses in quail (36–39), the viral attachment pattern of position 226 mutant viruses was determined by virus histochemistry using tracheal tissues obtained from Japanese quail (Fig. 6 and Table 2). Several distinct patterns of attachment were observed: in general, the N226, F226, Q226, and L226 (WF10) viruses attached more strongly to the tracheal epithelium than the M226 variant, which had moderate binding, while the H226, I226, V226, G226, and T226 variants bound with a mild intensity. In addition to differences in the intensity of binding, we observed differences in the footprint of attachment. While the I226, G226, V226, and H226 variants attached mildly in punctate and discrete areas near the base of the cilia, the M226 variant staining was more intense at the base of the ciliated tracheal epithelium. In contrast to the above-described variants, T226 variant binding was restricted to the basal structures underneath the tracheal respiratory epithelium. The Q226 virus attached abundantly to discrete areas on the ciliated epithelium, along with strong staining of the goblet cells for the Q226 virus. For the S226 variant, though binding to the cilia was moderate, the binding pattern resembled the one with the T226 virus. Similar attachment patterns were observed for the N226, F226, and L226 viruses, displaying abundant attachment to the cilia as well as moderate binding to the basement membrane of the trachea. Overall, the staining patterns in terms of the intensity and distribution among the different variants were, in general, consistent with the presence of both 2,3- and 2,6-linked SA receptors in the quail tracheal tissue (39).

**TABLE 2:** Summary of sialic acid specificity and virus histochemistry data for position 226 mutant viruses^*e*^

Variant virus^*a*^	Direct binding^*b*^				Binding to glycan array^*c*^							Quail VHC^*d*^
	3Fet-HRP	6Fet-HRP	3SLN	6SLN	2,3 L	2,3 O	2,3 N	L^x^	2,6 L	2,6 O	2,6 N	
I226	+/−	+/−	+	+	−	−	+++	−	++	++	++	+
S226	+/−	−	+	−	++	++	+++	++	−	−	+	++
T226	+/−	−	+	−	++	+	+++	+	−	−	+	+
M226	+	++	+	+	−	−	+/−	−	+	+	+++	++
H226	+	−	+	−	++	+/−	+++	−	−	−	−	+
N226	++	+	+	+	++	+	+++	+++	+++	++	+++	+++
F226	−	−	+	+	+	+	+++	+	+	++	+++	+++
V226	++	++	+	+	+/−	+/−	+	−	+++	++	+++	+
C226	++	−	+	−	+++	++	+++	+++	−	−	−	ND
G226	+	+	+	+	+++	++	+++	+++	+	++	+++	+
L226	−	++	+/−	+	+/−	+/−	+++	−	++++	++++	++++	+++
Q226	++	−	+	−	++++	++++	++++	−	−	−	−	+++

a Viruses on H9N2 backbone.

b Direct binding assays were scored as follows: +, binding to sialic acid substrate; −, no binding to sialic acid; +/−, low binding to both α2,3 and α2,6.

c Glycan microarray data were classified as follows: −, no binding; +, narrow binding; ++, moderate binding to some sugars; +++, strong binding to most/all sugars in the microarray.

d VHC, virus histochemistry scoring, which was as follows: +, low; ++, moderate; +++, intense; −, no binding; ND, not done.

e Fet, fetuin; HRP, horseradish peroxidase; L, linear; O, O linked; N, N linked; L^x^, sialyl-Lewis X.

### *In vivo* replication advantage of H9 variants.

**FIG 7:**
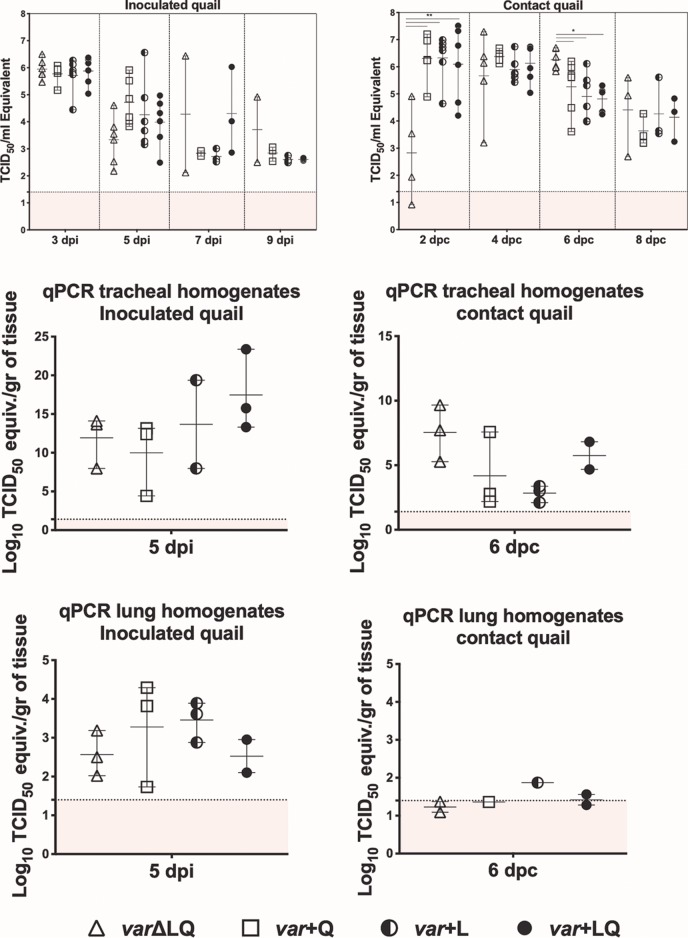
Replication and transmission phenotype of mutant viruses in quails. Quails were randomly divided into five groups; one group (inoculated with PBS) served as a control (not shown). At 4 to 5 weeks of age, 6 birds were inoculated intranasally, intratracheally, and cloacally with a mixture of viruses with or without Q226 and/or L226 at 10^6^ TCID_50_/ml per bird. Group 1 received only H9N2 variant viruses without L226 and Q226 (*var*ΔLQ), group 2 received variant viruses including the Q226 virus (*var*+Q), group 3 received variant viruses including the L226 virus (*var*+L), and the birds in group 4 received all variant viruses, including the Q226 and L226 viruses (*var*+LQ). On day 1 postinfection, 6 naive quails were introduced as direct-contact animals. (A, C, E) Viral shedding was quantified by quantitative reverse transcriptase PCR in tracheal swabs at 3, 5, 7, and 9 dpi and in tracheal and lung homogenates at 5 dpi for inoculated birds. (B, D, F) Viral shedding was quantified by quantitative reverse transcriptase PCR in tracheal swabs at 2, 4, 6, and 8 dpc and in tracheal and lung homogenates at 6 dpc for direct-contact birds. Statistically significant differences between the *var*ΔLQ group compared to the other groups are indicated. **, *P* < 0.001; *, *P* < 0.05.

Instead of testing each individual position 226 mutant virus for replication and transmission *in vivo*, we designed a competition study to investigate whether position 226 confers a relative fitness advantage. Thus, we inoculated young quail with mixtures of the mutant viruses on the H9N2 background. Groups of quails were designed based on the presence or absence of the L226 and Q226 viruses in the mixture used for inoculation. Quails were randomly assigned to 4 groups (*n* = 6/group). The birds in group 1 received an equal mix of the H9N2 viruses without L226 and Q226 (*var*ΔLQ). Group 2 (*var*+Q) birds received the H9N2 virus mix along with the Q226 virus, group 3 (*var*+L) birds received a similar mix with the L226 virus, and group 4 (*var*+LQ) birds received a mix of all the H9N2 variants and both the L226 and Q226 viruses. A fifth group of quail was mock inoculated with PBS as a negative control (not shown). At 2 days postinoculation (dpi), we introduced naive quail (*n* = 6/group) to monitor transmission. Over the course of 14 days, infected birds displayed minimal signs of disease, as has been previously reported (36, 38). By 3 dpi, all inoculated birds were positive, with all groups shedding similar levels of virus (∼10^6^ 50% tissue culture infectious dose [TCID_50_] equivalents/ml) (Fig. 7). Virus titers reduced by 7 dpi, with quails in the *var*+Q and *var*+L groups shedding an average of 10^3^ TCID_50_ equivalents/ml. In the *var*+LQ group, 2 birds shed between 10^4^ and 10^6^ TCID_50_ equivalents/ml, while the last bird in the group had titers similar to that of the birds in the *var*+Q and *var*+L groups. Only 2 birds in the *var*ΔLQ group were positive for virus by day 7, with one bird shedding up to 10^6^ TCID_50_ equivalents/ml. No significant differences in virus titers were observed among the different groups of inoculated birds.

Direct-contact quail in the *var*ΔLQ group showed delayed replication kinetics. While direct-contact quail in other groups shed an average of 10^6^ TCID_50_ equivalents/ml by 2 days postcontact (dpc), the average virus titer in tracheal swabs of *var*ΔLQ direct-contact birds was significantly less than that for all other contact groups (*P* < 0.001) (Fig. 7). By 6 dpc, the average virus titer shed by birds in the *var*ΔLQ group was significantly higher than that shed by birds in the other groups (*P* < 0.05). At 5 dpi and 6 dpc, 3 birds from each group were sacrificed for virus titration of the lungs and trachea. Viral titers were higher in the upper respiratory tract than in the lower respiratory tract in both inoculated and contact quails (Fig. 7). A trend toward higher peak titers in tracheal homogenates was observed in inoculated and contact quails, which showed the highest average peak titers in tracheal swabs at 5 dpi and 6 dpc, respectively.

**FIG 8:**
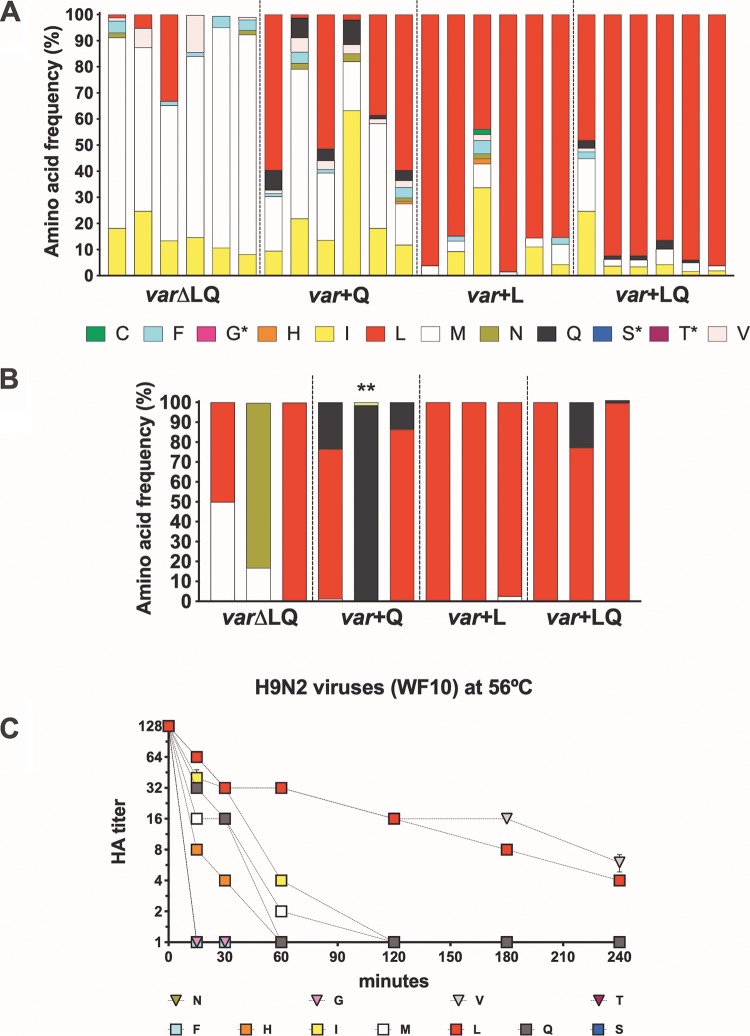
Frequency of amino acids present in tracheal swab samples of inoculated and contact quails and temperature stability of H9N2 variant viruses. The viruses in tracheal swab specimens collected at 3 dpi from inoculated quails (A) and 8 dpc from contact quails (B) were sequenced by NGS, and the amino acid frequency was analyzed using Geneious (version 10.2.3) software. Each bar represents a quail in the respective group, and each color represents a variant virus. Single asterisks refer to variant viruses included in the virus mix and not identified in tracheal swabs, while the double asterisk shows the K226 variant virus not included in the virus mix yet identified in contact quail. (C) Temperature stability of H9N2 variant viruses at 56°C. Variant viruses diluted to 128 HAU/50 μl were incubated at 56°C. Samples collected at 0, 15, 30, 60, 120, 180, and 240 min postincubation were used for hemagglutination assays. Treatment was carried out in quadruplicate. L, Q, M, F, I, S, H, N, V, C, T, and G correspond to the amino acid mutations at position 226 in the HA of H9N2 viruses. Naturally occurring amino acids previously found in H9 HA are shown as squares; others are shown as triangles. Differences were observed depending on which amino acid was present at position 226. By 1 h at 56°C, all H9N2 viruses had hemagglutination titers (HA titers) of less than 2 HAU, except viruses with aliphatic amino acids, V226, L226, and I226 viruses, as well as the C226 and Q226 viruses. After 4 h, only the viruses with V226 and L226 showed any hemagglutination titer and were the most stable viruses.

To determine what amino acids were predominant in inoculated quails and what amino acids were associated with contact transmission events, tracheal swab samples collected at 3 and 5 dpi (*n* = 6/group) and 4 and 8 dpc (*n* = 6 and *n* = 3/group, respectively) were subjected to whole-genome sequencing by NGS using the Illumina MiSeq platform. By 3 and 5 dpi, birds in the *var*+LQ group shed predominantly the L226 variant at a frequency of between 51 and 96% by 3 dpi (Fig. 8A). In one quail, the M226 and I226 variants made up 44% of virus shed by 3 dpi. Similar to the findings for this group, quails in the *var*+L group shed mainly the L226 variant (43 to 98% frequency). The I226 variant was also identified in one quail at a frequency of 33%. We observed a general increase in the proportion of the other variants being shed in the absence of the L226 variant in both the *var*+Q and *var*ΔLQ groups. However, the L226 variant was the consensus variant in 3 quails in the *var*+Q group by 3 dpi, while 2 birds shed the M226 variant, and the I226 variant was found in 1 quail. Without the commonly found L226 and Q226 variants, the M226 variant outcompeted all other variants in the *var*ΔLQ group, being shed at a frequency of between 52 and 84% in inoculated quails at 3 dpi. The V226, F226, and I226 variants were also identified in the *var*ΔLQ group. Though leucine was not a component of the mix in the *var*+Q and *var*ΔLQ groups, the L226 variant in the *var*+Q and *var*ΔLQ inoculated groups could have emerged from a single mutation in the second nucleotide of the codon encoding H226, the third nucleotide of the codon encoding F226, or the first nucleotide of the codon encoding either F226, I226, M226, or V226, which were present at lower frequencies in both groups.

For the contact quails, all birds in the *var*+L and *var*+LQ groups shed the L226 variant at 4 and 8 dpc (1 quail in the *var*+L group shed the M226 variant by 4 dpc) (Table 3; Fig. 8B), which was not surprising, given that most of the directly inoculated birds shed this variant by 3 dpi. In the *var*+Q group, the contact birds shed a mixed population of Q226 and L226 by 4 dpc, with 3 birds shedding mostly the L226 variant and the other 3 shedding Q226 viruses (Table 3); by 8 dpc the L226 variant was the most predominant variant, to the detriment of the Q226 variant (75 and 86%, respectively), while the last bird shed mainly the Q226 variant (Fig. 8B). In the *var*ΔLQ group, 3 different amino acid variants (I226, F226, and M226) were identified in the tracheal swabs of contact birds at 4 dpc (Table 3). By 8 dpc, one bird shed a mixture of the M226 and L226 variants, with the M226 variant being the consensus, while another bird shed mostly the L226 variant (99% frequency). A mixed population of N226 and M226 variants was present in the last quail, with the N226 variant being present at a frequency of 83%.

**TABLE 3:** Consensus variant analysis of 226 variant viruses in directly inoculated and direct-contact quail^*a*^

Group/day	Quail identifier(s)	Sequence									
		PB2	PB1	PA	HA^*b*^	NP	NA	M1	M2	NS1	NEP
*var*+LQ											
3 dpi	Q13–Q18				L226						
5 dpi	Q13				I226						
	Q14			K26E	L226						
	Q15			K26E	L226						
	Q16				L226						
	Q17				L226						
	Q18				L226						
4 dpc	Q55	H357Y		K26E	L226						
	Q56			K26E	N21T L226						
	Q57		D619N	K26E	L226						
	Q58			K26E	L226 D368E						
	Q59				L226						
	Q60		D619N	K26E	L226						
8 dpc	Q58			K26E	L226 D368E						
	Q59			K26E	L226						
	Q60		D619N	K26E	L226					N127H	
*var*+L											
3 dpi	Q1–Q6				L226						
5 dpi	Q1–Q6				L226						
4 dpc	Q43			K26E	L226					T91A	
	Q44				L226						
	Q45				L226						
	Q46				L226						
	Q47			K26E	L226						
	Q48	T303A	T204A		M226		N46K, ΔQ47_P50				
8 dpc	Q46			K26E	L226, A325S		A131S				
	Q47			K26E	L226						
	Q48			K26E	L226, A325S		N46K, ΔQ47_P50				
*var*+Q											
3 dpi	Q49				L226						
	Q50				M226						
	Q51				L226						
	Q52				I226						
	Q53				M226						
	Q54				L226						
5 dpi	Q49			K26E	L226						
	Q50				Q226						
	Q51			K26E	L226						
	Q52			K26E	M226						
	Q53			K26E	L226						
	Q54				L226						
4 dpc	Q31			K26E	L226						
	Q32			K26E	L226						
	Q33			K26E	L226						
	Q34			K26E, E327K	Q226						
	Q35			K26E	Q226						
	Q36			K26E	Q226						
8 dpc	Q34			K26E	L226						
	Q35			K26E	Q226						
	Q36			K26E	L226						
*var*ΔLQ											
3 dpi	Q25–Q30				M226						
5 dpi	Q25				L226						
	Q26				T135I P186T V226						
	Q27		N642V		F226 G228A						
	Q28		T204A		M226		M33I				
	Q29				M226						
	Q30				V226						
4 dpc	Q7				I226			N82S			
	Q8			K26E	M226						
	Q9				I226						
	Q10				M226		T383M				
	Q11		N642V		F226 G228A						
	Q12				F226						
8 dpc	Q10				M226		T383M				
	Q11		N642V		N226						
	Q12		T400I		L226						

a Only the consensus variant sequence is shown. A variant is identified to be different from the consensus sequence of wild-type WF10 virus. Minor variants are omitted from the table.

b Amino acids 226, 325, and 368 with H3 numbering (amino acids 216, 316, and 359 with H9 numbering).

## DISCUSSION

Receptor engagement is a critical step in influenza virus infection and is mediated by the HA. It is well-known that changes to amino acids in the receptor-binding site of HA modulate receptor recognition, which in turn influences host adaptation. Several studies have linked the changes at positions 226 and 228 (H3 numbering) to receptor preference. In the context of H2, H3, and H4 HA subtype viruses, the combination of Q226/G228 allows for avian-like α2-3SA recognition, while the L226/S228 combination favors human α2-6SA recognition (7, 9, 40, 41). In the particular case of H3 subtypes, position 226 appears to be under selective pressure, and most human H3 viruses have transitioned from L226 to V226 to I226. Interestingly, most human-origin swine H3 viruses possess V226, but it is not clear whether such an amino acid signature has promoted the more frequent human-to-swine interspecies transmission events seen in the past two decades. For the most part, H9 receptor studies have focused on multiple strains with more than one amino acid difference in the RBS site and have tested only naturally occurring mutations. From these studies, the contribution of position 226 in receptor preference in H9 viruses is well established, but it can also be modulated by other amino acids in the RBS (17, 42, 43). Analysis of the 4,826 unique H9 HA sequences from bird isolates available to date in the Influenza Research Database (IRD) revealed that ∼81% of isolates have L226, ∼18% have Q226, and the remaining ∼1% of the sequences carry M226, I226, H226, P226, S226, or F226 (Fig. 2A). The Q226L change has also been associated with improved direct contact and airborne transmission of H9N2 viruses in the ferret model (43, 44). Interestingly, the ferret data are consistent with the observation that despite the limited number of human cases, 85% of them (*n* = 17 out 20) have been associated with the L226 variant (Fig. 2A). It could be argued that the proportion of human cases is simply a reflection of the prevalence of the L226 variant in poultry, but such an argument does not explain why pigs have been more susceptible to the Q226 variant (∼55%, *n* = 25 out of 45) than to the L226 variant (42%, *n* = 19). The differences in susceptibility to the L226 versus Q226 variants in humans versus pigs can perhaps be better explained by their different SA receptor distribution profiles (45). Our results reveal great flexibility at this position, identifying a diverse set of amino acids that can be accommodated. From the 15 different variants identified in the H9N2 and/or H9N1 subtype configuration, 7 of them were associated with amino acids present in at least one natural isolate identified in IRD (F226, H226, I226, L226, M226, Q226, and S226). The rest of the variants (A226, G226, N226, R226, T226, V226, Y226) are yet to be found in natural isolates of H9 viruses, while one (C226) is yet to be found in any influenza virus HA subtype. We did not obtain a variant with P226, which is represented by a single isolate in the database, or variants with an acidic amino acid (D226, E226), a basic amino acid (K226), or an indole side chain (W226). It was surprising to observe that some of the most favored variants from the H9N2 and H9N1 virus libraries corresponded to amino acids that have yet to be found in nature (N226, *n* = 17 overall) or that are uncommon (H226, *n* = 13 overall) in the background of the H9 HA (Fig. 2A). It is unlikely that the lack of selection for the L226 variant in the WF10 background was because of a lack of representation in the _nnn226_H9PCR library: the exact same _nnn226_H9PCR library was used to generate the _nnn_H9N1 virus library, resulting in the L226 variant as the most prominent variant in the virus pool (*n* = 5; *n* = 12 overall). Like the L226 variant, the Q226 variant was obtained only when paired with the N1 NA in the PR8 virus background. These results indicate that *in vitro* and *in vivo* fitness does not necessarily correlate and question whether some of the variants observed in natural isolates could indeed be artifacts of the method used for virus growth, something that could be easily ascertained by sequencing the virus directly from the field sample.

Using cell avidity, solid-phase binding, glycan array analyses, and tissue-binding profiles, we determined the receptor preference of these single-amino-acid variants. In terms of relative α2-3SA and α2-6SA preference, the two ends of the spectrum are represented by the Q226 variant, which binds strongly to sugars containing α2-3SAs, while the L226 variant preferentially binds sugars containing α2-6SAs. These observations are consistent with previous reports (17, 42). The binding profiles of the Q226 and L226 variants were unaffected by the paired NA subtypes, indicating the limited influence of the latter in receptor recognition. Similar to previous studies with H7N9 and H3N2 viruses (46, 47), there was an overall preference for N-linked glycans by all viruses tested. N-linked glycans are known to be predominant in the human and ferret glycomes (48, 49). We isolated viruses with hydrophobic amino acids V226 and I226, residues which are commonly found in H3 viruses and are associated with α2-6SA binding (50). In the H9 variants, these viruses behaved similarly, binding α2-6SAs and α2-3SAs in solid-phase assays, but with more α2-6SA preference in glycan arrays. Though the amino acids involved with receptor specificity in H1 influenza viruses do not include position 226, R226 has been found in an H1 virus following adaptation to egg that allowed for α2-3SA recognition, an observation consistent with the findings for the R226 H9N1 virus, which showed an avidity for α2-3SAs receptors (data not shown) (51). Furthermore, we observed a pattern of polar amino acid residues (C226, H226, S226, and T226) recognizing the α2-3SAs almost exclusively, while hydrophobic amino acid residues (G226, F226, and M226) were able to interact with both α2-3SA and α2-6SA sugars. The exception to this observation was with the N226 variant (a polar residue), which was able to bind to both sugars. On the crystal structure of the A/swine/Hong Kong/9/98 (H9N2) virus HA, L226 makes nonpolar interactions with the C-6 atom of the SA (52). With the exception of N226, all the dual-binding viruses contain hydrophobic amino acids and are capable of making van der Waals interactions that allow recognition of SAs in the α2-6 conformation. Other amino acids in the RBS have been implicated in the receptor-binding preference of H9 viruses. In a recent study, an A190V mutation in an H9N2 virus was identified as being able to expand SA recognition by widening the RBS pocket (53), while in H3 viruses, E190D increased α2-6SA recognition (54). All variant viruses in this study possessed an E190 residue, which is fairly common in influenza viruses of avian origin. In addition to the position 190 mutation, the I155T, H183N, and G228S mutations have also been associated with changes in receptor preference (55). It has been suggested that the I155T mutation plays an important role in H9N2 virus binding to human-like SA receptors (43). However, the viruses described here have distinct patterns of α2-3SA and/or α2-6SA receptor binding, depending on the amino acid residue at position 226, despite carrying the T155 profile, suggesting that the contribution of the I155T change may be minimal for human-like receptor recognition or that its contribution is context dependent. It would be of interest to test whether strains carrying the I155 profile could regain an α2-6SA preference by introducing the Q226L mutation.

The *in vitro* data showed a similar fitness for all of the mutants, as they replicated to similar titers by 72 hpi in avian (except the S226 and I226 variants) and mammalian (except the I226 variant) cell lines. Results from the *in vivo* competition studies in quails, however, indicate that there are bottlenecks affecting the virus phenotype *in vivo* that cannot be recapitulated by *in vitro* systems. Due to limitations in the number of animal studies that could be performed, we decided to ascertain the relative fitness advantage of the position 226 variant viruses in an *in vivo* competition model. Undoubtedly, the L226 virus showed an *in vivo* advantage in the quail model. Interestingly, the L226 variant was the most thermostable virus among the H9N2 variants (along with the V226 variant; Fig. 8C), which is also consistent with the increased body temperature of birds (∼40°C). L226 was the predominant virus in inoculated and contact quails in the *var*+L and *var*+LQ groups, where this variant was included in the infection mix. This is corroborated by the presence of L226 in ∼80% of naturally occurring isolates. Interestingly, in the *var*+Q group, where Q226 but not L226 was included in the virus mix, the L226 virus was ultimately selected in both inoculated and direct-contact birds, which is consistent with previous studies that have shown that position 226 is under selective pressure (50, 56). The M226 virus was the predominant variant in all directly inoculated quails in the *var*ΔLQ group at 3 dpi. However, other variants were also capable of replicating and transmitting in the absence of the Q226 and L226 variants in the mix, most notably, the I226 variant, the F226 variant, and the non-naturally occurring V226 and N226 variants. The I226 variant was also among the most common variants in all inoculated groups, which is in sharp contrast to its relatively lower replication kinetics *in vitro*. Nevertheless, the I226 variant was outcompeted by the M226, L226, and Q226 viruses in the direct-contact birds, suggesting a lower fitness *in vivo* compared to that of the most prominent transmitted viruses, consistent with their relatively lower frequency in poultry. A single nucleotide change in the M226 codon (ATG) can lead to the emergence of the L226 codon (CTG). Similarly, the L226 variant detected in birds in the *var*+Q group could have easily emerged from a single nucleotide change in the Q226 codon (CAA) to produce the L226 codon (CTA). It is noteworthy that the N226 variant was detected at a high frequency in one direct-contact quail by 8 dpc, despite having the lowest frequency (∼4%) of all the identified variants in the *var*ΔLQ group by 3 dpi. Unexpectedly, we found the variant K226, which was not part of the initial inoculum and which represented ∼1.5% of the variants, in 1 contact quail in the *var*+Q group at 8 dpc. The significance of the N226 and K226 variants for transmission and their potential emergence in nature remain to be determined. Despite generating NGS information for 2 different time points postinfection in both inoculated and direct-contact quails, we should note that our data represent a snapshot of a dynamic replication and transmission event. We cannot exclude the possibility of a more complex mixed population of variants initiating infection/transmission, although our data suggest the progressive dominant effect of one variant over the course of infection. Additional mutations were identified throughout the viral genome depending on the quail sample analyzed (Table 3). Most of these mutations appear to be random mutation events that do not remain fixed in the virus population. The exception is the PA K26E mutation, which appeared consistently in many of the samples and which became the dominant amino acid signature in samples from direct-contact quail, suggesting a potential fitness advantage. Potentially relevant mutations were identified in the HA, particularly the G228A mutation associated with the F226 variant. Although we cannot assign any specific biological significance to the G228A mutation in the H9 HA, it suggests some level of flexibility that is worth exploring in more detail in future studies. We must note that the G228S mutation in the H3 HA played a major role in the emergence of the pandemic H3 virus by allowing better recognition of human-like SA receptors. In the HA, we also identified the mutations A325S and D368E in some virus samples following transmission (Table 3). The A325S (A316S, H9 numbering) mutation in the cleavage site identified in 2 quails (*var*+L group) is characteristic of recent BJ/94-like H9N2 isolates in China (57). Interestingly, this mutation was identified along with mutations in NA either as a single amino acid mutation, A131S, or as a 4-amino-acid deletion (deletion of amino acids 47 to 50) of a contact quail in the *var*+L group; the latter combination has been linked with increased virulence in chickens and mice (58). Additional synonymous and nonsynonymous mutations were identified (Table 3), but further analyses are beyond the scope of the present report.

This study identified amino acids that have yet to be seen in H9 viruses and that have the ability to expand the SA preference of H9 viruses. This not only is important for surveillance efforts to identify viruses of greater pandemic concern but also can be harnessed in areas such as vaccine production. Approaches that improve vaccine production *in vitro* but that decrease HA fitness *in vivo* without sacrificing its immunogenicity are important in the context of major efforts toward universal influenza virus vaccines based on live attenuated approaches. The use of RBS mutant vaccine viruses could perhaps preclude the current issues accompanying the growth of H3N2 vaccine viruses in eggs. RBS variant vaccine viruses could also complement the safety profile of live virus vaccines, as they are likely to be less fit than viruses with the wild-type segment. In summary, we have shown the plasticity of amino acid 226 in the HA of the H9 subtype influenza virus and demonstrated the significant effects on cell avidity and receptor binding *in vitro*, in tissues, and *in vivo*. Further studies like this are necessary to provide a comprehensive mutational map of the amino acid changes that can modulate receptor recognition of influenza viruses.

## MATERIALS AND METHODS

### Ethics statement.

All animal studies adhered to the Institutional Animal Care and Use Committee (IACUC) guidebook of the Office of Laboratory Animal Welfare (59) and PHS policy on the humane care and of use of laboratory animals. Studies were conducted under animal biosafety level 2 (ABSL-2) containment and approved by the Institutional Animal Care and Use Committee (IACUC) of the University of Georgia (protocol A201506-026-Y3-A5). Animals were humanely euthanized following guidelines approved by the American Veterinary Medical Association (AVMA).

### Cells.

Madin-Darby canine kidney (MDCK) and human embryonic kidney 293T cells were a kind gift from Robert Webster, St. Jude Children’s Research Hospital, Memphis, TN. Chicken fibroblast-derived (DF1) cells were a kind gift from Siba Samal, University of Maryland, College Park, MD. Cells were maintained in Dulbecco’s modified Eagle’s medium (DMEM; Sigma-Aldrich, St. Louis, MO) containing 10% fetal bovine serum (FBS; Sigma-Aldrich, St. Louis, MO), 1% antibiotic-antimycotic (ATB-ATM; Sigma-Aldrich, St. Louis, MO), 1% l-glutamine, and 2.5% HEPES (Sigma-Aldrich, St. Louis, MO). Cells were cultured at 37°C in a humidified incubator under 5% CO_2_.

### Generation of reverse genetics-ready _226_HA PCR library.

The full-length cDNA copy of the HA gene segment from A/guinea fowl/Hong Kong/WF10/1999 (H9N2) virus (WF10) was split into two overlapping fragments and cloned into two separate plasmids. The plasmid pDPAO1-767 contains nucleotides 1 to 767 of the H9 HA gene segment preceded by the 5′ end sequence corresponding to the mouse RNA polymerase 1 (pol 1) terminator (t1). The plasmid pDPAO738-1742 contains nucleotides 738 to 1742 of the H9 HA segment immediately upstream of the human RNA polymerase 1 (pol 1) promoter (Fig. 1). The _226_HA PCR library was generated by focusing on the codon encoding amino acid 226 using two different approaches. In the first approach, a PCR product was produced from pDPAO1-767 using the primer set t1FragFwd and WF10-HA713-757_nnn_ (primer sequences are available upon request). The resulting *5′-t1_nnn_HA757-3′* PCR product included the t1 terminator sequence and the HA fragment with the NNN codon at position 226. In the second approach, an equivalent PCR product, *5′-t1_equi_HA757-3′*, was generated from pDPAO1-767 using the primer t1FragFwd and 50 pmol/μl of a primer mix containing 20 primers designed to introduce every possible amino acid at position 226. The second half of the HA was amplified from pDPAO738-1742 using the set of primers WF10 HA 738-770 and hPol1Rev to generate the PCR product *5′-HA738pol1-3′* that included the second half of the HA followed by the pol 1 promoter sequence. The full-length reverse genetics-ready HA PCR librarie, _nnn226_H9PCR and _equi226_H9PCR were obtained by overlapping PCR using either the *5′-t1_nnn_HA757-3′* or the *5′-t1_equi_HA757-3′* PCR fragment, respectively, along with the *5′-HA738pol1-3′* PCR fragment and the primer pair t1FragFwd and hPol1Rev. The 50-μl PCR mixture contained 10 ng of each PCR product, 25 μl of the PCR master mix, 50 pmol/μl of each primer, and 1.5 μl of dimethyl sulfoxide. PCR amplification was done using a Phusion high-fidelity PCR master mix with GC buffer (New England Biolabs, Ipswich, MA) under the following cycling parameters: 98°C for 30 s; 98°C for 8 s, 56°C for 1 min, and 72°C for 3 min for 30 cycles; and 72°C for 10 min (Fig. 1A).

### Generation of _nnn226_H9N2, _nnn226_H9N1, _equi226_H9N2, and _equi226_H9N1 virus libraries.

Independent virus rescue experiments were performed with the _nnn226_H9PCR and _equi226_H9PCR amplicon libraries, as previously described (29, 60). To generate the _nnn226_H9N2 and _equi226_H9N2 virus libraries, the corresponding PCR amplicons were cotransfected along with 7 reverse genetics plasmids encoding the rest of the WF10 genome (Fig. 1B). To generate the _nnn226_H9N1 and _equi226_H9N1 virus libraries, the PCR amplicons were cotransfected along with 7 reverse genetics plasmids encoding the PB2, PB1, PA, NP, NA, M, and NS gene segments of the laboratory-adapted strain A/Puerto Rico/8/1934 (H1N1) (PR8). For transfection, cocultures of MDCK and 293T cells were seeded in each well of a 6-well plate overnight at 37°C. On the following day, 1 μg of each of the 7 plasmids of either WF10 or PR8 and 1 μg of PCR amplicon were mixed with 16 μl of the TransIT-LT1 transfection reagent (Mirus Bio LLC, Madison, WI), and the mixture was incubated for 45 min. After 45 min, the MDCK/293T cells were overlaid with the transfection mixture and incubated at 37°C for 24 h. At 24 h posttransfection (hpt), the transfection mixture was replaced with fresh Opti-MEM I medium (Life Technologies, Carlsbad, CA) containing 1 μg/ml of tosylsulfonyl phenylalanyl chloromethyl ketone (TPCK)-treated trypsin (Worthington Biochemicals, Lakewood, NJ) and 1% ATB-ATM. Supernatants containing rescued viruses were collected at 96 hpt.

### Isolation and identification of individual virus variants and growth of virus stocks.

Single virus variants in the virus libraries were isolated by limiting dilution assays as previously described (61). Briefly, MDCK cells (2 × 10^4^ cells/well) in a 96-well plate were infected with 8 serial 10-fold dilutions of the rescued virus library in Opti-MEM I medium (Life Technologies, Carlsbad, CA) containing 1 μg/ml of TPCK-treated trypsin and 1% ATB-ATM. The variant strains were produced starting from the _nnn226_H9N2 virus library (*n* = 12), the _nnn226_H9N1 virus library (*n* = 12), the _equi226_H9N2 virus library (*n* = 24), and the _equi226_H9N1 virus library (*n* = 24). After 72 h of incubation at 37°C, virus supernatants were collected from wells infected with the most diluted sample displaying a cytopathic effect (CPE). This process was repeated once, followed by Sanger sequencing to determine the amino acid at position 226 in HA. A third round of limiting dilution was carried out for samples yet to resolve at position 226 after the second limiting dilution. The viruses were further expanded in MDCK cells, the stocks were aliquoted, and the aliquots were stored at −80°C until use. The virus stocks were titrated by use of the 50% tissue culture infectious dose (TCID_50_) in MDCK cells, and titers were determined by the Reed and Muench method (62).

### Generation of quail antisera and hemagglutination and HI assays.

Three-week-old Japanese quails (Coturnix japonica) were infected with 10^6^ TCID_50_ of WF10 or the M226 virus and boosted at 14 dpi with the respective inactivated virus containing 1:1 (vol/vol) Montanide as an adjuvant. The quails were bled for serum collection at 14 days after the boost, and the antisera collected were used in hemagglutination inhibition (HI) assays. Standard hemagglutination assays were performed using either 1% horse red blood cells (RBCs) (Lampire Biologicals, Pipersville, PA) or 0.5% chicken RBCs or 0.5% turkey RBCs (Poultry Diagnostic Research Center, Athens, GA) and expressed in hemagglutination units (HAU) as previously described (63). HI assays were performed as previously described (63) using the H9 variant viruses as antigens and quail sera generated against the WF10 (H9N2) virus and the M226 virus.

### Preparation of viral RNA and cDNA.

Viral RNA and cDNA from variant viruses were prepared as previously described (29). Total RNA was extracted from variant viruses using an RNeasy kit (Qiagen, Valencia, CA) following the manufacturer’s protocol. To obtain cDNA, reverse transcription was carried out using avian myeloblastosis virus reverse transcriptase (Promega, Madison, WI) and primer Uni12 (AGCAAAAGCAAGG).

### *In vitro* growth kinetics assays.

MDCK and DF1 cells were infected with wild-type and variant H9 viruses at an MOI of 0.01 in 6-well plates for 15 min at 4°C and 45 min at 37°C. Following 3 washes with phosphate-buffered saline (PBS) to remove any unbound virus, MDCK cells were overlaid with 2 ml Opti-MEM I (Life Technologies, Carlsbad, CA) medium containing ATB-ATM (Sigma-Aldrich, St. Louis, MO) and 1 μg/ml TPCK-treated trypsin. For infected DF1 cells, TPCK-treated trypsin was replaced with 5% allantoic fluid. At 6, 12, 24, 48, and 72 h postinfection (hpi), the supernatant was collected and stored at −80°C until viral titration in MDCK cells. The viral titer was determined by measurement of the TCID_50_ using the Reed and Muench method (62).

### Glycan array analysis.

Glycan array analysis was performed using an *N*-hydroxysuccinimide (NHS) ester-coated glass microarray slide containing six replicates of 128 diverse sialic acid-containing glycans, including terminal sequences, and intact N-linked and O-linked glycans found on mammalian and avian glycoproteins and glycolipids. The virus stock was amplified in MDCK cells, clarified by centrifugation, and inactivated using β-propiolactone at a final concentration of 0.1%. Inactivated virus samples were then purified by ultracentrifugation at 18,000 rpm for 2 h at 4°C, resuspended in PBS–5% glycerol, and stored at −80°C. Whole H9 influenza virus samples were diluted to 256 HAU/50 μl (final concentration) in PBS containing 3% bovine serum albumin (BSA) and incubated on the array surface for 1 h at room temperature (RT) in a humidity-controlled chamber. After 1 h, the slides were washed in PBS and incubated with a virus-specific anti-H9 mouse monoclonal antibody, AIV-H9-HA 3G8 (64) (derived from mouse ascitic fluid), diluted 1:200 in PBS, 3% BSA for a further 1 h. The slides were washed in PBS and incubated for a final 1 h in goat anti-mouse IgG-Alexa Fluor Plus 488 (final concentration, 10 μg/ml; catalog number A32723; Thermo Fisher Scientific) diluted in PBS, 3% BSA. The slides were washed twice in PBS and in distilled water (dH_2_O) and then dried prior to detection. Slide scanning to detect bound virus was conducted using an InnoScan-n1100AL (Innopsys, Carbonne, France) fluorescent microarray scanner. The fluorescent signal intensity was measured using Mapix software (Innopsys, Carbonne, France), and the mean intensity minus the mean background for 4 replicate spots was calculated. A complete list of the glycans on the array is presented in Table S1 in the supplemental material.

### Cell binding assays.

The cell binding assay was performed as previously described (65, 66). Chicken RBCs were treated for 1 h at 37°C with 2-fold serial dilutions of neuraminidase from Clostridium perfringens (New England Biolabs, Ipswich, MA) to remove sialic acids. Following neuraminidase treatment, the RBCs were washed twice with PBS and resuspended to a 1% solution with PBS. Then, 50 μl of 1% RBCs was incubated with 50 μl of virus (8 HAU) at RT for 45 min to determine the hemagglutination titer.

### Solid-phase binding assays.

Solid-phase direct binding assays using sialylglycopolymers were performed as previously described (15, 62). Briefly, 96-well fetuin-coated flat-bottom plates (Grenier Bio-One, Monroe, NC) were incubated overnight at 4°C with 128 HAU of purified variant virus in 50 μl 0.02 M Tris-buffered saline (TBS), pH 7.2 to 7.4. The plates were subsequently blocked with blocking solution (BS; PBS containing 0.1% neuraminidase-treated bovine serum albumin [BSA-NA]) for 2 h at room temperature (RT). After blocking, the plates were washed 3 times with ice-cold washing solution (WS; PBS containing 0.02% Tween 80) and incubated with 50 μl 2-fold serial dilution of high-molecular-weight polyacrylamide (PAA)-linked biotinylated sialylglycopolymer-3SLN-PAA-biotin or 6SLN-PAA-biotin (GlycoTech, Gaithersburg, MD) in reaction solution (RS; PBS containing 0.02% Tween 80, 0.1% BSA-NA, and 2 μM oseltamivir carboxylate) at 4°C for 1 h. Subsequently, the wells were washed 5 times with ice-cold WS and incubated with a 1:1,000 dilution of streptavidin-horseradish peroxidase (Thermo Fisher, Rockford, IL) for 1 h at 4°C. After incubation, the plates were washed 5 times with WS before adding 100 μl of freshly prepared substrate solution (SS; 0.01% 3,3′,5 5′-tetramethylbenzidine in 0.05 M sodium acetate with 0.03% H_2_O_2_). The reactions were stopped with 3% H_2_SO_4_ after 30 min, unless otherwise stated_._ Absorbance readings were obtained at 450 nm using a Victor X3 plate reader (PerkinElmer, Waltham, MA). Assays were carried out twice, and viruses were tested in duplicate.

### Virus histochemistry with quail tracheal tissues.

Tracheal tissues from influenza virus-negative 4- to 6-week old Japanese quail were used for virus histochemical studies. Virus histochemistry was performed as previously described using fluorescein isothiocyanate (FITC)-labeled viruses (72). Briefly, tissue culture-grown variant viruses were clarified by low-speed centrifugation and concentrated by ultracentrifugation at 28,000 rpm for 2 h at 4°C prior to resuspension in PBS. Concentrated viruses were labeled with FITC by mixing equal volumes of virus and 0.1 mg/ml of freshly prepared FITC in 0.5 M bicarbonate buffer at pH 9.5, and excess label was removed by dialysis in PBS. Formalin-fixed, paraffin-embedded tissues were deparaffinized using xylene, rehydrated with graded alcohol, and subsequently incubated with FITC-labeled viruses (100 HAU/50 μl) at 4°C overnight in a humidified chamber. Following washing with 0.2 M Tris-HCl, 0.1 M NaCl, 0.5% Tween 20 (TNT) buffer, FITC was detected using a peroxidase-labeled rabbit anti-FITC antibody (Dako, Glostrup, Denmark). To enhance detection, the signal was amplified using a Tyramide signal amplification system (Perkin Elmer, Akron, OH) according to the manufacturer’s protocol. 3-Amino-9-ethyl-carbazole (AEC; Sigma-Aldrich, St. Louis, MO) was used to reveal the peroxidase, and counterstaining was done with Mayer hematoxylin. Viral attachment was seen as red staining. The intensity of binding to the surface epithelium of the trachea was scored as none (−), low (+), moderate (++), and intense (+++) (Table 2).

### *In vivo* competition study.

Four groups of 4- to 5-week-old Japanese quail (*n* = 6/group) were used for the *in vivo* competition study. Quail eggs obtained from the College of Veterinary Medicine, University of Georgia, were hatched at the Poultry Diagnostic and Research Center, University of Georgia. A week before virus inoculation, quails were bled and confirmed to be seronegative for IAV exposure. Quails were inoculated with 1 ml of virus mix (0.25 ml administered via the trachea and nares and 0.5 ml via the cloaca) containing a homogenous mixture of variant viruses with or without L226 or Q226 virus. In group 1 (*var*ΔLQ), birds were inoculated with 10^6^ TCID_50_ virus mix containing the following 10 variant (*var*) viruses on the WF10 backbone: I226, S226, T226, M226, H226, N226, F226, V226, C226, and G226 viruses. Group 2 (*var*+Q) birds received all 10 *var* viruses and the Q226 virus, while group 3 birds (*var*+L) were infected with the 10 *var* viruses and the L226 (WF10) virus. Group 4 (*var***+**LQ) birds were infected with a mix of all 10 *var* viruses along with the L226 and Q226 viruses (*var***+**LQ). A final group of 6 birds served as the negative control receiving 1 ml PBS through the same routes (group 5 [PBS]; not shown). At 1 day postinfection (dpi), naive quail (*n* = 6/group) were introduced as direct contacts to determine transmission. At 5 dpi and 6 dpc, 3 quail from each group of directly inoculated and contact quail, respectively, were randomly selected and sacrificed for virus titration in tissues. Tracheal and cloacal swab specimens were collected daily from each bird until 14 dpi. Swabs were suspended in 1 ml 3.7% brain heart infusion (BHI) medium (Becton, Dickinson, Sparks, MD) containing 10,000 U penicillin, 10 mg streptomycin, and 25 μl amphotericin B and stored at −80°C until use in virus titrations.

### Quantification of virus shedding in quail samples.

Virus RNA was isolated from tracheal swab samples using a MagMAX-96 AI/ND viral RNA isolation kit (Thermo Fisher Scientific, Waltham, MA) following the manufacturer’s instructions and eluted in 50 μl of nuclease-free molecular-grade water. A one-step quantitative PCR (qPCR) based on the avian influenza virus matrix gene as a surrogate of virus shedding was carried out using the primer/probe set previously described (67). The qPCR was performed in a LightCycler 480 real-time PCR instrument (Roche Diagnostics, Rotkreuz, Switzerland) using a LightCycler 480 RNA master hydrolysis probe kit (Roche Life Science, Mannheim, Germany) in a final reaction volume of 20 μl. Each reaction mixture contained 1× LightCycler 480 probes master mix, 0.5 μM forward and reverse primers, 0.3 μM probe, and 5 μl of RNA. The qPCR cycling conditions ran at 61°C for 10 min and a denaturation step of 95°C for 30 s, followed by 45 cycles of amplification at 95°C for 10 s, 60°C for 20 s, and 72°C for 1 s, with a final cooling step at 40°C for 10 s. A standard curve was generated using 10-fold serial dilutions of a WF10 virus stock of known titer to correlate qPCR crossing point (*C_p_*) values with the amount of virus shed from each bird, as previously described (68).

### Sequencing.

Standard Sanger sequencing was performed on the full-length _nnn226_HA and _equi226_HA PCR products prior to transfection and on HA from all *var* virus stocks. Sequences were generated using specific primers, a BigDye Terminator (v3.1) cycle sequencing kit (Applied Biosystems, Carlsbad, CA), and a 3100 genetic analyzer (Applied Biosystems, Carlsbad, CA) according to the manufacturer’s instructions. For whole-genome sequencing of viral stock and tracheal swab samples, RNA was extracted using an RNeasy minikit (Qiagen, Valencia, CA) or a MagNA Pure LC RNA isolation kit (Roche Life Science, Mannheim, Germany). Isolated virus RNA served as the template in a one-step reverse transcriptase PCR for multisegment, whole-genome amplification, as previously described (69). Amplicon sequence libraries were prepared using a Nextera XT DNA library preparation kit (Illumina, San Diego, CA) according to the manufacturer’s protocol. Barcoded libraries were multiplexed and sequenced on a high-throughput Illumina MiSeq sequencing platform in a paired-end 150-nucleotide run format. *De novo* genome assembly was performed as described previously (69).

### Virus thermal stability assays.

H9 variant viruses were diluted to 128 HAU/50 μl and then incubated on a heat block at 56°C for various times (0, 15, 30, 60, 120, 180, and 240 min). Subsequently, HA assays were carried out on heat-treated viruses in quadruplicate.

### Database analysis of HA sequences.

H9 HA sequences were obtained from the Influenza Research Database (IRD) (70) through the web site at http://www.fludb.org (accessed on 18 March 2018). The amino acid frequency at position 226 was then analyzed using the protein sequence variant analysis tool provided by the IRD. Whole-genome sequences were then mapped to the WF10 reference sequence using Geneious (version 10.2.3) software (71).

### Statistical analysis.

Data analyses were performed using GraphPad Prism (version 7) software (GraphPad Software Inc., San Diego, CA). For multiple comparisons, either one-way or two-way analysis of variance (ANOVA) was performed, followed by a *post hoc* Tukey test. A *P* value below 0.05 (*P* < 0.05) was considered significant.

### Accession number(s).

The Illumina data are available through NCBI’s Short Read Archive (https://www.ncbi.nlm.nih.gov/sra) under accession numbers SAMN10530904 through SAMN10530939 and BioProject accession number PRJNA508785.

## Supplementary Material

Supplemental file 1
