# Supplementary material for: Flexibility In Vitro of Amino Acid 226 in the Receptor-Binding Site of an H9 Subtype Influenza A Virus and Its Effect In Vivo on Virus Replication, Tropism, and Transmission
Source: J Virol. 2019 Mar 5;93(6):e02011-18. doi: 10.1128/JVI.02011-18 (PMC6401463; doi:10.1128/JVI.02011-18)
Supplement: Supplemental file 1 [file JVI.02011-18-s0001.pdf]

Supplementary Table 1. List of glycan structures present on the array

Glycan #	Common Name	Structure
1	Galβ(1-4)GlcNAcβ-ethyl-NH2	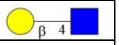
2	Galβ(1-4)GlcNAcβ(1-3)Galβ(1-3)GalNAca-Thr-NH2	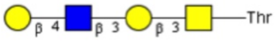
3	Galβ(1-4)GlcNAcβ(1-6)[Galβ(1-3)]-GalNAca-Thr-NH2	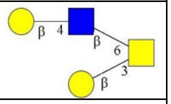
4	Galβ(1-4)GlcNAcβ(1-3)GalNAca-Thr-NH2	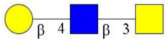
5	Galβ(1-4)GlcNAcβ(1-3)[Galβ(1-4)GlcNAcβ(1-6)]-GalNAca-Thr-NH2	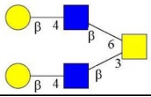
6	Galβ(1-4)GlcNAcβ(1-6)GalNAca-Thr-NH2	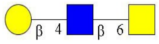
7	Galβ(1-4)GlcNAcβ(1-2)Mana (1-3)[Galβ(1-4)GlcNAcβ(1-2)Mana(1-6)]-Manβ(1-4)GlcNAcβ(1-4)GlcNAcβ-Asn-NH2	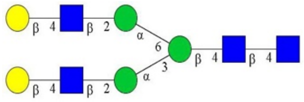
8	Galβ(1-4)GlcNAcβ(1-2)Mana(1-3)[Galβ(1-4)GlcNAcβ(1-2)Mana(1-6)]-Manβ(1-4)GlcNAcβ(1-4)[Fuca(1-6)]-GlcNAcβ-Asn-Ser-Thr-NH2	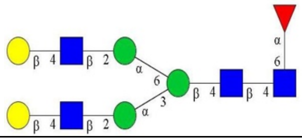
9	Galβ(1-4)GlcNAcβ(1-2)Mana(1-3){Galβ(1-4)GlcNAcβ(1-2)[Galβ(1-4)GlcNAcβ(1-2)]-Mana(1-6)}-Manβ(1-4)GlcNAcβ(1-4)GlcNAcβ-Asn-Lys-NH2	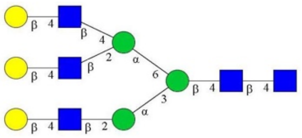
10	Galβ(1-4)GlcNAcβ(1-2)Mana(1-3){Galβ(1-4)GlcNAcβ(1-2)[Galβ(1-4)GlcNAcβ(1-2)]-Mana(1-6)}-Manβ(1-4)GlcNAcβ(1-4)[Fuca(1-6)]-GlcNAcβ-(Lys-Val-Ala)Asn-Lys-Thr-NH2	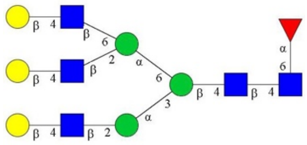
11	NeuAca(2-3)Galβ(1-4)6-O-sulfo-GlcNAcβ-propyl-NH2	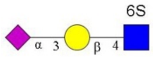
12	NeuAca(2-3)Galβ(1-4)[Fuca(1-3)]-6-O-sulfo-GlcNAcβ-propyl-NH2	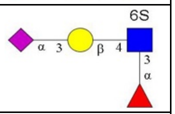
13	NeuAca(2-3)6-O-sulfo-Galβ(1-4)GlcNAcβ-ethyl-NH2	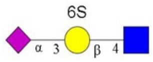
14	NeuAca(2-3)6-O-sulfo-Galβ(1-4)[Fuca(1-3)]-GlcNAcβ-propyl-NH2	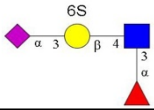
15	NeuAca(2-3)Galβ(1-3)6-O-sulfo-GlcNAcβ-propyl-NH2	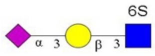
16	NeuAca(2-3)Galβ(1-4)Glcβ-ethyl-NH2	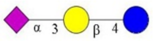
17	NeuAca(2-3)Galβ(1-4)GlcNAcβ-ethyl-NH2	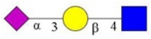
18	NeuAca(2-3)Galβ(1-4)GlcNAcβ(1-3)Galβ(1-4)GlcNAcβ-ethyl-NH2	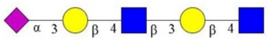
19	NeuAca(2-3)Galβ(1-4)GlcNAcβ(1-3)Galβ(1-4)GlcNAcβ(1-3)Galβ(1-4)GlcNAcβ-ethyl-NH2	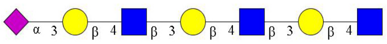

Glycan #	Common Name	Structure
**20**	NeuAca(2-3)GalNAcβ(1-4)GlcNAcβ-ethyl-NH2	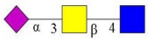
**21**	NeuAca(2-3)Galβ(1-3)GlcNAcβ-ethyl-NH2	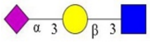
**22**	NeuAca(2-3)Galβ(1-3)GlcNAcβ(1-3)Galβ(1-4)GlcNAcβ-ethyl-NH2	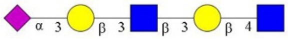
**23**	NeuAca(2-3)Galβ(1-3)GlcNAcβ(1-3)Galβ(1-3)GlcNAcβ-ethyl-NH2	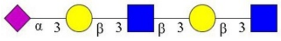
**24**	NeuAca(2-3)Galβ(1-3)GalNAcβ(1-3)Gala(1-4)Galβ(1-4)Glcβ-ethyl-NH2	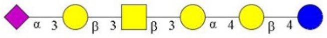
**25**	NeuAca(2-3)Galβ(1-3)GalNAca-Thr-NH2	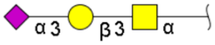
**26**	NeuAca(2-3)Galβ(1-4)GlcNAcβ(1-3)Galβ(1-3)GalNAca-Thr-NH2	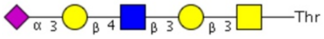
**27**	NeuAca(2-3)Galβ(1-4)GlcNAcβ(1-3)Galβ(1-4)GlcNAcβ(1-3)Galβ(1-3)GalNAca-Thr-NH2	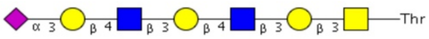
**28**	NeuAca(2-3)Galβ(1-4)GlcNAcβ(1-3)Galβ(1-4)GlcNAcβ(1-3)Galβ(1-4)GlcNAcβ(1-3)Galβ(1-3)GalNAca-Thr-NH2	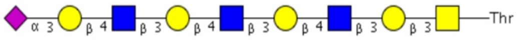
**29**	NeuAca(2-3)Galβ(1-4)GlcNAcβ(1-3)Galβ(1-4)GlcNAcβ(1-3)Galβ(1-4)GlcNAcβ(1-3)Galβ(1-4)GlcNAcβ(1-3)Galβ(1-3)GalNAca-Thr-NH2	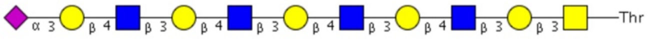
**30**	NeuAca(2-3)Galβ(1-4)GlcNAcβ(1-3)Galβ(1-4)GlcNAcβ(1-3)Galβ(1-4)GlcNAcβ(1-3)Galβ(1-4)GlcNAcβ(1-3)Galβ(1-4)GlcNAcβ(1-3)Galβ(1-3)GalNAca-Thr-NH2	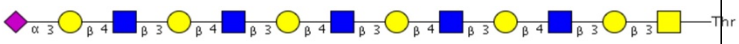
**31**	NeuAca(2-3)Galβ(1-4)GlcNAcβ(1-6)[Galβ(1-3)]-GalNAca-Thr-NH2	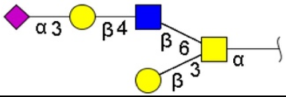
**32**	NeuAca(2-3)Galβ(1-4)GlcNAcβ(1-3)Galβ(1-4)GlcNAcβ(1-6)[Galβ(1-3)]-GalNAca-Thr-NH2	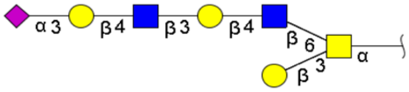
**33**	NeuAca(2-3)Galβ(1-4)GlcNAcβ(1-3)Galβ(1-4)GlcNAcβ(1-3)Galβ(1-4)GlcNAcβ(1-6)[Galβ(1-3)]-GalNAca-Thr-NH2	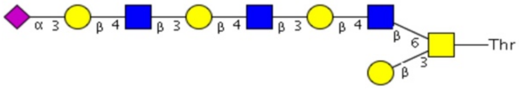
**34**	NeuAca(2-3)Galβ(1-4)GlcNAcβ(1-3)Galβ(1-4)GlcNAcβ(1-3)Galβ(1-4)GlcNAcβ(1-3)Galβ(1-4)GlcNAcβ(1-6)[Galβ(1-3)]-GalNAca-Thr-NH2	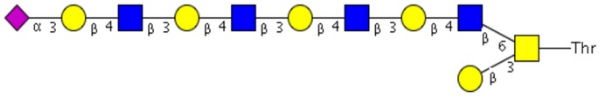
**35**	NeuAca(2-3)Galβ(1-4)GlcNAcβ(1-3)Galβ(1-4)GlcNAcβ(1-3)Galβ(1-4)GlcNAcβ(1-3)Galβ(1-4)GlcNAcβ(1-3)Galβ(1-4)GlcNAcβ(1-6)[Galβ(1-3)]-GalNAca-Thr-NH2	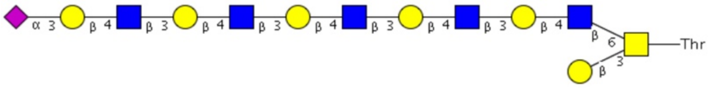
**36**	NeuAca(2-3)Galβ(1-4)GlcNAcβ(1-3)Galβ(1-4)GlcNAcβ(1-3)Galβ(1-4)GlcNAcβ(1-3)Galβ(1-4)GlcNAcβ(1-6)[NeuAca(2-3)Galβ(1-4)GlcNAcβ(1-3)Galβ(1-4)GlcNAcβ(1-3)Galβ(1-4)GlcNAcβ(1-3)Galβ(1-3)]-GalNAca-Thr-NH2	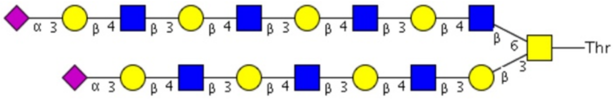

Glycan #	Common Name	Structure
**37**	NeuAca(2-3)Galβ(1-4)GlcNAcβ(1-3)Galβ(1-4)GlcNAcβ(1-3)Galβ(1-4)GlcNAcβ(1-3)Galβ(1-4)GlcNAcβ(1-3)Galβ(1-4)GlcNAcβ(1-6)[NeuAca(2-3)Galβ(1-4)GlcNAcβ(1-3)Galβ(1-4)GlcNAcβ(1-3)Galβ(1-4)GlcNAcβ(1-3)Galβ(1-4)GlcNAcβ(1-3)Galβ(1-3)]-GalNAca-Thr-NH2	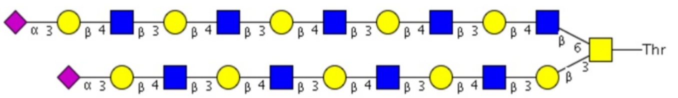
**38**	NeuAca(2-3)Galβ(1-4)GlcNAcβ(1-3)GalNAca-Thr-NH2	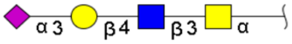
**39**	NeuAca(2-3)Galβ(1-4)GlcNAcβ(1-3)Galβ(1-4)GlcNAcβ(1-3)GalNAca-Thr-NH2	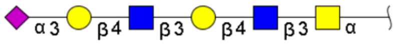
**40**	NeuAca(2-3)Galβ(1-4)GlcNAcβ(1-3)Galβ(1-4)GlcNAcβ(1-3)Galβ(1-4)GlcNAcβ(1-3)GalNAca-Thr-NH2	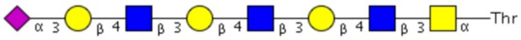
**41**	NeuAca(2-3)Galβ(1-4)GlcNAcβ(1-3)Galβ(1-4)GlcNAcβ(1-3)Galβ(1-4)GlcNAcβ(1-3)Galβ(1-4)GlcNAcβ(1-3)GalNAca-Thr-NH2	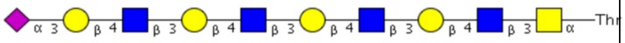
**42**	NeuAca(2-3)Galβ(1-4)GlcNAcβ(1-3)Galβ(1-4)GlcNAcβ(1-3)Galβ(1-4)GlcNAcβ(1-3)Galβ(1-4)GlcNAcβ(1-3)Galβ(1-4)GlcNAcβ(1-3)GalNAca-Thr-NH2	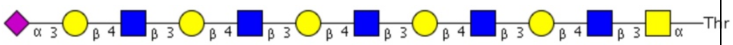
**43**	NeuAca(2-3)Galβ(1-4)GlcNAcβ(1-3)[NeuAca(2-3)Galβ(1-4)GlcNAcβ(1-6)]-GalNAca-Thr-NH2	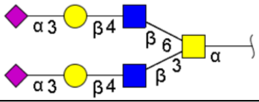
**44**	NeuAca(2-3)Galβ(1-4)GlcNAcβ(1-3)Galβ(1-4)GlcNAcβ(1-3)[NeuAca(2-3)Galβ(1-4)GlcNAcβ(1-3)Galβ(1-4)GlcNAcβ(1-6)]-GalNAca-Thr-NH2	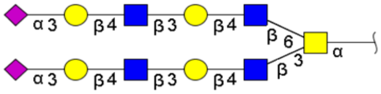
**45**	NeuAca(2-3)Galβ(1-4)GlcNAcβ(1-3)Galβ(1-4)GlcNAcβ(1-3)Galβ(1-4)GlcNAcβ(1-3)[NeuAca(2-3)Galβ(1-4)GlcNAcβ(1-3)Galβ(1-4)GlcNAcβ(1-3)Galβ(1-4)GlcNAcβ(1-6)]-GalNAca-Thr-NH2	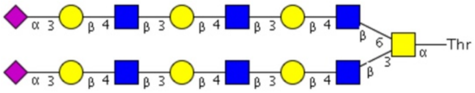
**46**	NeuAca(2-3)Galβ(1-4)GlcNAcβ(1-3)Galβ(1-4)GlcNAcβ(1-3)Galβ(1-4)GlcNAcβ(1-3)Galβ(1-4)GlcNAcβ(1-3)[NeuAca(2-3)Galβ(1-4)GlcNAcβ(1-3)Galβ(1-4)GlcNAcβ(1-3)Galβ(1-4)GlcNAcβ(1-3)Galβ(1-4)GlcNAcβ(1-6)]-GalNAca-Thr-NH2	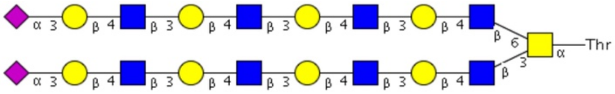
**47**	NeuAca(2-3)Galβ(1-4)GlcNAcβ(1-3)Galβ(1-4)GlcNAcβ(1-3)Galβ(1-4)GlcNAcβ(1-3)Galβ(1-4)GlcNAcβ(1-3)Galβ(1-4)GlcNAcβ(1-3)[NeuAca(2-3)Galβ(1-4)GlcNAcβ(1-3)Galβ(1-4)GlcNAcβ(1-3)Galβ(1-4)GlcNAcβ(1-3)Galβ(1-4)GlcNAcβ(1-3)Galβ(1-4)GlcNAcβ(1-6)]-GalNAca-Thr-NH2	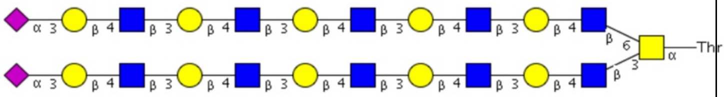
**48**	NeuAca(2-3)Galβ(1-4)GlcNAcβ(1-3)Galβ(1-4)GlcNAcβ(1-3)Galβ(1-4)GlcNAcβ(1-3)Galβ(1-4)GlcNAcβ(1-6)GalNAca-Thr-NH2	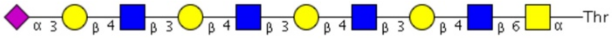

Glycan #	Common Name	Structure
**49**	NeuAca(2-3)Galβ(1-4)GlcNAcβ(1-3)Galβ(1-4)GlcNAcβ(1-3)Galβ(1-4)GlcNAcβ(1-3)Galβ(1-4)GlcNAcβ(1-3)Galβ(1-4)GlcNAcβ(1-6)GalNAca-Thr-NH2	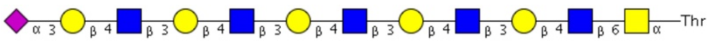
**50**	NeuAca(2-3)Galβ(1-3)GlcNAcβ(1-3)Galβ(1-4)GlcNAcβ(1-6)[NeuAca(2-3)Galβ(1-3)GlcNAcβ(1-3)] Galβ(1-4)GlcNAcβ-ethyl-NH2	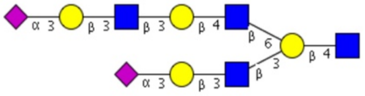
**51**	NeuAca(2-3)Galβ(1-4)GlcNAcβ(1-3)Galβ(1-4)GlcNAcβ(1-3)Galβ(1-4)GlcNAcβ(1-6)[NeuAca(2-3)Galβ(1-4)GlcNAcβ(1-3)Galβ(1-4)GlcNAcβ(1-3)] Galβ(1-4)GlcNAcβ-ethyl-NH2	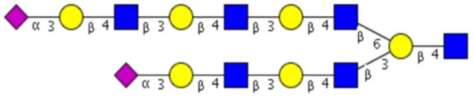
**52**	NeuAca(2-3)Galβ(1-4)GlcNAcβ(1-2)Mana(1-3)[NeuAca(2-3)Galβ(1-4)GlcNAcβ(1-2)Mana(1-6)]-Manβ(1-4)GlcNAcβ(1-4)GlcNAcβ-Asn-NH2	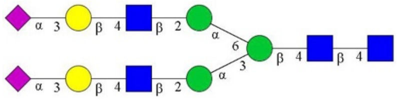
**53**	NeuAca(2-3)Galβ(1-4)GlcNAcβ(1-3)Galβ(1-4)GlcNAcβ(1-2)Mana(1-3)[NeuAca(2-3)Galβ(1-4)GlcNAcβ(1-3)Galβ(1-4)GlcNAcβ(1-2)Mana(1-6)]-Manβ(1-4)GlcNAcβ(1-4)GlcNAcβ-Asn-NH2	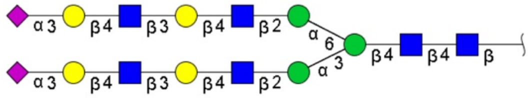
**54**	NeuAca(2-3)Galβ(1-4)GlcNAcβ(1-3)Galβ(1-4)GlcNAcβ(1-3)Galβ(1-4)GlcNAcβ(1-2)Mana(1-3)[NeuAca(2-3)Galβ(1-4)GlcNAcβ(1-3)Galβ(1-4)GlcNAcβ(1-3)Galβ(1-4)GlcNAcβ(1-2)Mana(1-6)]-Manβ(1-4)GlcNAcβ(1-4)GlcNAcβ-Asn-NH2	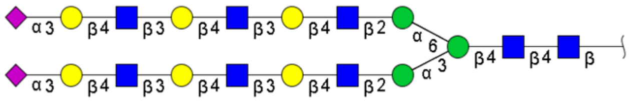
**55**	NeuAca(2-3)Galβ(1-4)GlcNAcβ(1-3)Galβ(1-4)GlcNAcβ(1-2)Mana(1-3)[NeuAca(2-3)Galβ(1-4)GlcNAcβ(1-3)Galβ(1-4)GlcNAcβ(1-2)Mana(1-6)]-Manβ(1-4)GlcNAcβ(1-4)GlcNAcβ-(Lys-Val-Ala)Asn-Lys-Thr-NH2	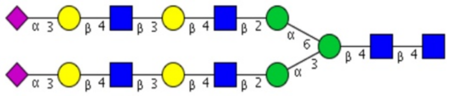
**56**	NeuAca(2-3)Galβ(1-4)GlcNAcβ(1-3)Galβ(1-4)GlcNAcβ(1-3)Galβ(1-4)GlcNAcβ(1-2)Mana(1-3)[NeuAca(2-3)Galβ(1-4)GlcNAcβ(1-3)Galβ(1-4)GlcNAcβ(1-3)Galβ(1-4)GlcNAcβ(1-2)Manα(1-6)]-Manβ(1-4)GlcNAcβ(1-4)GlcNAcβ-(Lys-Val-Ala)Asn-Lys-Thr-NH2	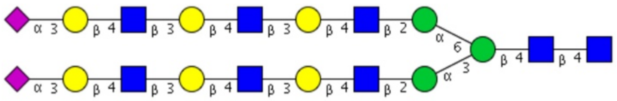
**57**	NeuAca(2-3)Galβ(1-4)GlcNAcβ(1-3)Galβ(1-4)GlcNAcβ(1-3)Galβ(1-4)GlcNAcβ(1-3)Galβ(1-4)GlcNAcβ(1-2)Mana(1-3)[NeuAca(2-3)Galβ(1-4)GlcNAcβ(1-3)Galβ(1-4)GlcNAcβ(1-3)Galβ(1-4)GlcNAcβ(1-3)Galβ(1-4)GlcNAcβ(1-2)Manα(1-6)]-Manβ(1-4)GlcNAcβ(1-4)GlcNAcβ-(Lys-Val-Ala)Asn-Lys-Thr-NH2	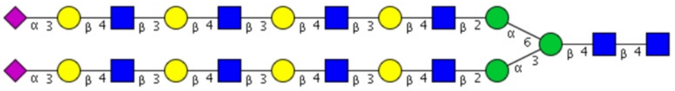

Glycan #	Common Name	Structure
**58**	NeuAca(2-3)Galβ(1-4)GlcNAcβ(1-3)Galβ(1-4)GlcNAcβ(1-3)Galβ(1-4)GlcNAcβ(1-3)Galβ(1-4)GlcNAcβ(1-3)Galβ(1-4)GlcNAcβ(1-2)Mana(1-3)[NeuAca(2-3)Galβ(1-4)GlcNAcβ(1-3)Galβ(1-4)GlcNAcβ(1-3)Galβ(1-4)GlcNAcβ(1-3)Galβ(1-4)GlcNAcβ(1-2)Mana(1-6)]-Manβ(1-4)GlcNAcβ(1-4)GlcNAcβ-(Lys-Val-Ala)Asn-Lys-Thr-NH2	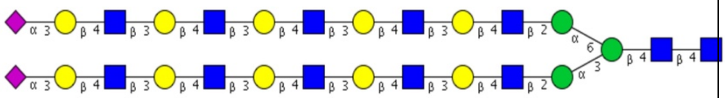
**59**	NeuAca(2-3)Galβ(1-4)GlcNAcβ(1-3)Galβ(1-4)GlcNAcβ(1-3)Galβ(1-4)GlcNAcβ(1-2)Mana(1-3)[NeuAca(2-3)Galβ(1-4)GlcNAcβ(1-3)Galβ(1-4)GlcNAcβ(1-3)Galβ(1-4)GlcNAcβ(1-2)Mana(1-6)]-Manβ(1-4)GlcNAcβ(1-4)[Fucα(1-6)]-GlcNAcβ-(Lys-Val-Ala)Asn-Lys-Thr-NH2	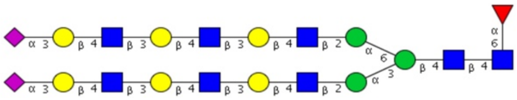
**60**	NeuAca(2-3)Galβ(1-4)GlcNAcβ(1-3)Galβ(1-4)GlcNAcβ(1-3)Galβ(1-4)GlcNAcβ(1-3)Galβ(1-4)GlcNAcβ(1-2)Mana(1-3)[NeuAcα(2-3)Galβ(1-4)GlcNAcβ(1-3)Galβ(1-4)GlcNAcβ(1-3)Galβ(1-4)GlcNAcβ(1-3)Galβ(1-4)GlcNAcβ(1-2)Manα(1-6)]-Manβ(1-4)GlcNAcβ(1-4)[Fucα(1-6)]-GlcNAcβ-(Lys-Val-Ala)Asn-Lys-Thr-NH2	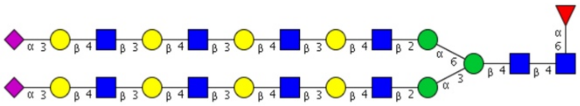
**61**	NeuAca(2-3)Galβ(1-4)GlcNAcβ(1-3)Galβ(1-4)GlcNAcβ(1-2)Mana(1-3){NeuAca(2-3)Galβ(1-4)GlcNAcβ(1-3)Galβ(1-4)GlcNAcβ(1-2)[NeuAca(2-3)Galβ(1-4)GlcNAcβ(1-3)Galβ(1-4)GlcNAcβ(1-6)Manα(1-6)]}-Manβ(1-4)GlcNAcβ(1-4)GlcNAcβ-(Lys-Val-Ala)Asn-Lys-Thr-NH2	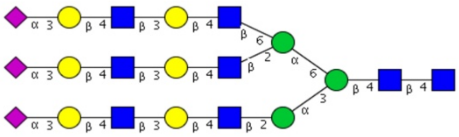
**62**	NeuAca(2-3)Galβ(1-4)GlcNAcβ(1-3)Galβ(1-4)GlcNAcβ(1-3)Galβ(1-4)GlcNAcβ(1-2)Manα(1-3){NeuAca(2-3)Galβ(1-4)GlcNAcβ(1-3)Galβ(1-4)GlcNAcβ(1-3)Galβ(1-4)GlcNAcβ(1-2)[NeuAca(2-3)Galβ(1-4)GlcNAcβ(1-3)Galβ(1-4)GlcNAcβ(1-3)Galβ(1-4)GlcNAcβ(1-6)Mana(1-6)]}-Manβ(1-4)GlcNAcβ(1-4)GlcNAcβ-(Lys-Val-Ala)Asn-Lys-Thr-NH2	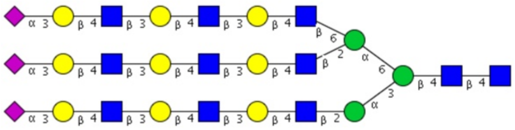
**63**	NeuAca(2-3)Galβ(1-4)GlcNAcβ(1-3)Galβ(1-4)GlcNAcβ(1-2)Mana(1-3){NeuAca(2-3)Galβ(1-4)GlcNAcβ(1-3)Galβ(1-4)GlcNAcβ(1-2)[NeuAca(2-3)Galβ(1-4)GlcNAcβ(1-3)Galβ(1-4)GlcNAcβ(1-6)Mana(1-6)]}-Manβ(1-4)GlcNAcβ(1-4)[Fuca(1-6)]-GlcNAcβ-(Lys-Val-Ala)Asn-Lys-Thr-NH2	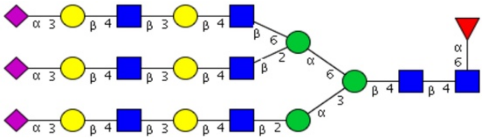

Glycan #	Common Name	Structure
**64**	NeuAca(2-3)Galβ(1-4)GlcNAcβ(1-3)Galβ(1-4)GlcNAcβ(1-3)Galβ(1-4)GlcNAcβ(1-2)Mana(1-3){NeuAca(2-3)Galβ(1-4)GlcNAcβ(1-3)Galβ(1-4)GlcNAcβ(1-3)Galβ(1-4)GlcNAcβ(1-2)[NeuAca(2-3)Galβ(1-4)GlcNAcβ(1-3)Galβ(1-4)GlcNAcβ(1-3)Galβ(1-4)GlcNAcβ(1-6)Mana(1-6)]}-Manβ(1-4)GlcNAcβ(1-4)[Fuca(1-6)]-GlcNAcβ-(Lys-Val-Ala)Asn-Lys-Thr-NH2	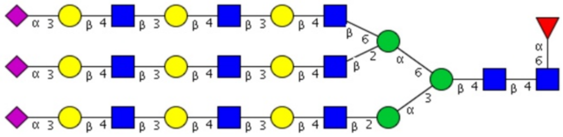
**65**	Gn/3'SLN/3'SLN-TriN	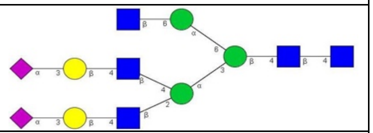
**66**	NeuAca(2-3)[GalNAcβ(1-4)]-Galβ(1-4)GlcNAcβ-ethyl-NH2	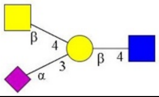
**67**	NeuAca(2-3)[GalNAcβ(1-4)]-Galβ(1-4)Glcβ-ethyl-NH2	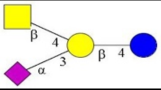
**68**	Galβ(1-3)GalNAcβ(1-4)[NeuAca(2-3)]-Galβ(1-4)Glcβ-ethyl-NH2	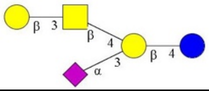
**69**	NeuAca(2-3)Galβ(1-4)[Fuca(1-3)]-GlcNAcβ-propyl-NH2	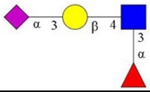
**70**	NeuAca(2-3)Galβ(1-3)[Fuca(1-4)]-GlcNAcβ(1-3)Galβ(1-4)[Fuca(1-3)]-GlcNAcβ-ethyl-NH2	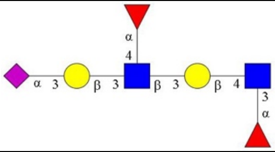
**71**	NeuAca(2-3)Galβ(1-4)[Fuca(1-3)]-GlcNAcβ(1-3)Galβ(1-4)[Fuca(1-3)]-GlcNAcβ-ethyl-NH2	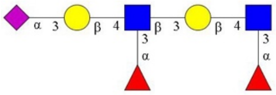
**72**	NeuAca(2-3)Galβ(1-4)[Fuca(1-3)]-GlcNAcβ(1-3)Galβ(1-4)[Fuca(1-3)]-GlcNAcβ(1-3)Galβ(1-4)[Fuca(1-3)]-GlcNAcβ-ethyl-NH2	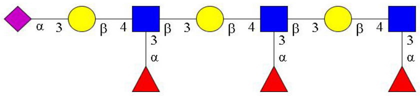
**73**	NeuAca(2-3)Galβ(1-4)[Fuca(1-3)]-GlcNAcβ(1-3)Galβ(1-4)[Fuca(1-3)]-GlcNAcβ(1-3)Galβ(1-4)[Fuca(1-3)]-GlcNAcβ(1-3)Galβ(1-3)GalNAca-Thr-NH2	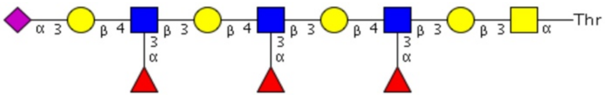
**74**	NeuAca(2-3)Galβ(1-4)[Fuca(1-3)]-GlcNAcβ(1-3)Galβ(1-4)[Fuca(1-3)]-GlcNAcβ(1-3)Galβ(1-4)[Fuca(1-3)]-GlcNAcβ(1-3)GalNAca-Thr-NH2	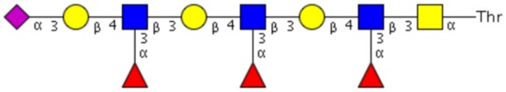
**75**	NeuAca(2-3)Galβ(1-4)[Fuca(1-3)]-GlcNAcβ(1-3)Galβ(1-4)[Fuca(1-3)]-GlcNAcβ(1-3)Galβ(1-4)[Fuca(1-3)]-GlcNAcβ(1-3)[NeuAca(2-3)Galβ(1-4)[Fuca(1-3)]-GlcNAcβ(1-3)Galβ(1-4)[Fuca(1-3)]-GlcNAcβ(1-3)Galβ(1-4)[Fuca(1-3)]-GlcNAcβ(1-6)]-GalNAca-Thr-NH2	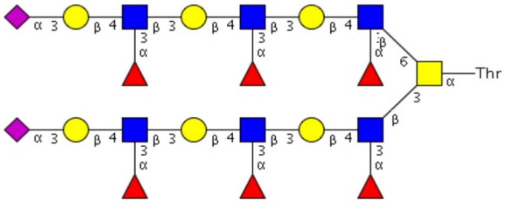

Glycan #	Common Name	Structure
**76**	NeuAca(2-3)Galβ(1-4)[Fuca(1-3)]-GlcNAcβ(1-2)Mana(1-3)[NeuAca(2-3)Galβ(1-4)[Fuca(1-3)]-GlcNAcβ(1-2)Mana(1-6)]-Manβ(1-4)GlcNAcβ(1-4)GlcNAcβ-(Lys-Val-Ala)Asn-(Lys-Thr)NH2	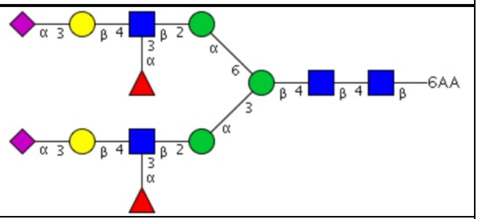
**77**	NeuAca(2-6)Galb(1-4)(6S)GlcNacb-ethyl-NH2	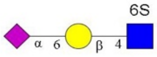
**78**	NeuAca(2-6)Galβ(1-4)6-O-sulfo-GlcNAcβ-propyl-NH2	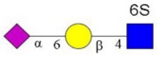
**79**	NeuAca(2-6)Galβ(1-4)Glcβ-ethyl-NH2	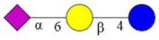
**80**	NeuAca(2-6)Galβ(1-4)GlcNAcβ-ethyl-NH2	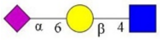
**81**	NeuAca(2-6)Galβ(1-4)GlcNAcβ(1-3)Galβ(1-4)GlcNAcβ-ethyl-NH2	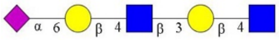
**82**	NeuAca(2-6)Galβ(1-4)GlcNAcβ(1-3)Galβ(1-4)GlcNAcβ(1-3)Galβ(1-4)GlcNAcβ-ethyl-NH2	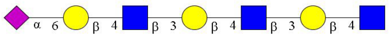
**83**	NeuAca(2-6)GalNAcβ(1-4)GlcNAcβ-ethyl-NH2	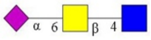
**84**	NeuAca(2-6)Galβ(1-4)GlcNAcβ(1-3)Galβ(1-3)GalNAca-Thr-NH2	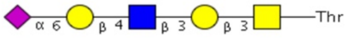
**85**	NeuAca(2-6)Galβ(1-4)GlcNAcβ(1-3)Galβ(1-4)GlcNAcβ(1-3)Galβ(1-3)GalNAca-Thr-NH2	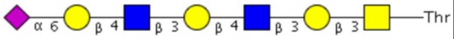
**86**	NeuAca(2-6)Galβ(1-4)GlcNAcβ(1-3)Galβ(1-4)GlcNAcβ(1-3)Galβ(1-4)GlcNAcβ(1-3)Galβ(1-3)GalNAca-Thr-NH2	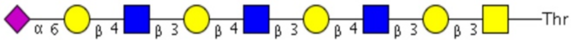
**87**	NeuAca(2-6)Galβ(1-4)GlcNAcβ(1-3)Galβ(1-4)GlcNAcβ(1-3)Galβ(1-4)GlcNAcβ(1-3)Galβ(1-4)GlcNAcβ(1-3)Galβ(1-3)GalNAca-Thr-NH2	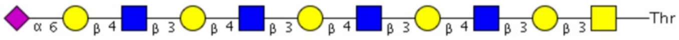
**88**	NeuAca(2-6)Galβ(1-4)GlcNAcβ(1-3)Galβ(1-4)GlcNAcβ(1-3)Galβ(1-4)GlcNAcβ(1-3)Galβ(1-4)GlcNAcβ(1-3)Galβ(1-4)GlcNAcβ(1-3)Galβ(1-3)GalNAca-Thr-NH2	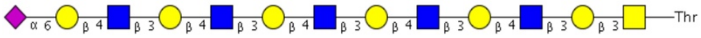
**89**	NeuAca(2-6)Galβ(1-4)GlcNAcβ(1-6)[Galβ(1-3)]-GalNAca-Thr-NH2	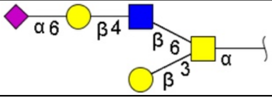
**90**	NeuAca(2-6)Galβ(1-4)GlcNAcβ(1-3)Galβ(1-4)GlcNAcβ(1-6)[Galβ(1-3)]-GalNAca-Thr-NH2	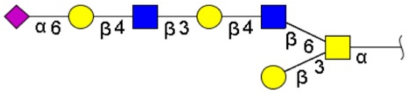
**91**	NeuAca(2-6)Galβ(1-4)GlcNAcβ(1-3)Galβ(1-4)GlcNAcβ(1-3)Galβ(1-4)GlcNAcβ(1-6)[Galβ(1-3)]-GalNAca-Thr-NH2	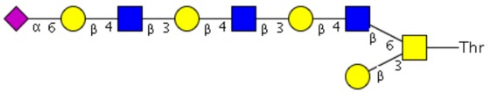
**92**	NeuAca(2-6)Galβ(1-4)GlcNAcβ(1-3)Galβ(1-4)GlcNAcβ(1-3)Galβ(1-4)GlcNAcβ(1-3)Galβ(1-4)GlcNAcβ(1-6)[Galβ(1-3)]-GalNAca-Thr-NH2	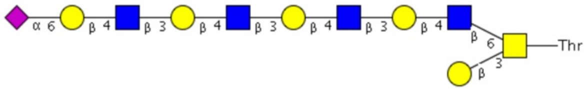

Glycan #	Common Name	Structure
**93**	NeuAca(2-6)Galβ(1-4)GlcNAcβ(1-3)Galβ(1-4)GlcNAcβ(1-3)Galβ(1-4)GlcNAcβ(1-3)Galβ(1-4)GlcNAcβ(1-3)Galβ(1-4)GlcNAcβ(1-6)[Galβ(1-3)]-GalNAca-Thr-NH2	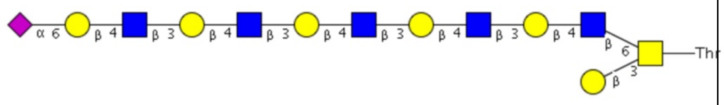
**94**	NeuAca(2-6)Galβ(1-4)GlcNAcβ(1-3)Galβ(1-4)GlcNAcβ(1-3)Galβ(1-4)GlcNAcβ(1-3)Galβ(1-4)GlcNAcβ(1-6)[NeuAca(2-6)Galβ(1-4)GlcNAcβ(1-3)Galβ(1-4)GlcNAcβ(1-3)Galβ(1-4)GlcNAcβ(1-3)Galβ(1-3)]-GalNAca-Thr-NH2	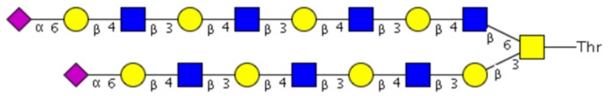
**95**	NeuAca(2-6)Galβ(1-4)GlcNAcβ(1-3)Galβ(1-4)GlcNAcβ(1-3)Galβ(1-4)GlcNAcβ(1-3)Galβ(1-4)GlcNAcβ(1-3)Galβ(1-4)GlcNAcβ(1-6)[NeuAcα(2-6)Galβ(1-4)GlcNAcβ(1-3)Galβ(1-4)GlcNAcβ(1-3)Galβ(1-4)GlcNAcβ(1-3)Galβ(1-4)GlcNAcβ(1-3)Galβ(1-3)]-GalNAca-Thr-NH2	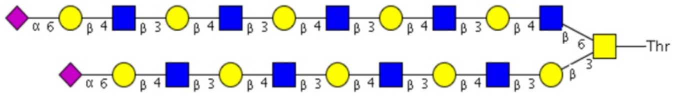
**96**	NeuAca(2-6)Galβ(1-4)GlcNAcβ(1-3)GalNAca-Thr-NH2	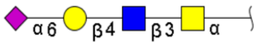
**97**	NeuAca(2-6)Galβ(1-4)GlcNAcβ(1-3)Galβ(1-4)GlcNAcβ(1-3)GalNAca-Thr-NH2	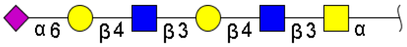
**98**	NeuAca(2-6)Galβ(1-4)GlcNAcβ(1-3)Galβ(1-4)GlcNAcβ(1-3)Galβ(1-4)GlcNAcβ(1-3)GalNAca-Thr-NH2	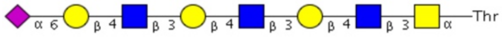
**99**	NeuAca(2-6)Galβ(1-4)GlcNAcβ(1-3)Galβ(1-4)GlcNAcβ(1-3)Galβ(1-4)GlcNAcβ(1-3)Galβ(1-4)GlcNAcβ(1-3)GalNAca-Thr-NH2	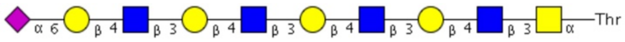
**100**	NeuAca(2-6)Galβ(1-4)GlcNAcβ(1-3)Galβ(1-4)GlcNAcβ(1-3)Galβ(1-4)GlcNAcβ(1-3)Galβ(1-4)GlcNAcβ(1-3)Galβ(1-4)GlcNAcβ(1-3)GalNAca-Thr-NH2	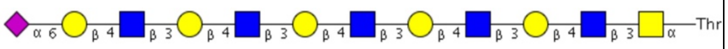
**101**	NeuAca(2-6)Galβ(1-4)GlcNAcβ(1-3)[NeuAca(2-6)Galβ(1-4)GlcNAcβ(1-6)]-GalNAca-Thr-NH2	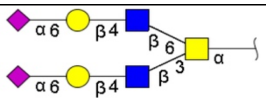
**102**	NeuAca(2-6)Galβ(1-4)GlcNAcβ(1-3)Galβ(1-4)GlcNAcβ(1-3)[NeuAca(2-6)Galβ(1-4)GlcNAcβ(1-3)Galβ(1-4)GlcNAcβ(1-6)]-GalNAca-Thr-NH2	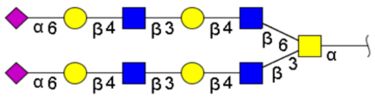
**103**	NeuAca(2-6)Galβ(1-4)GlcNAcβ(1-3)Galβ(1-4)GlcNAcβ(1-3)Galβ(1-4)GlcNAcβ(1-3)[NeuAca(2-6)Galβ(1-4)GlcNAcβ(1-3)Galβ(1-4)GlcNAcβ(1-3)Galβ(1-4)GlcNAcβ(1-6)]-GalNAca-Thr-NH2	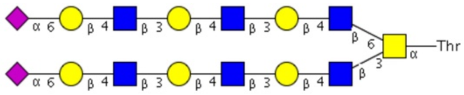
**104**	NeuAca(2-6)Galβ(1-4)GlcNAcβ(1-3)Galβ(1-4)GlcNAcβ(1-3)Galβ(1-4)GlcNAcβ(1-3)Galβ(1-4)GlcNAcβ(1-3)[NeuAca(2-6)Galβ(1-4)GlcNAcβ(1-3)Galβ(1-4)GlcNAcβ(1-3)Galβ(1-4)GlcNAcβ(1-3)Galβ(1-4)GlcNAcβ(1-6)]-GalNAca-Thr-NH2	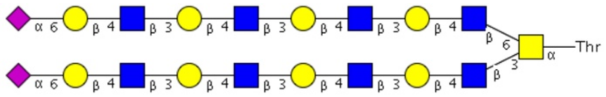

Glycan #	Common Name	Structure
**105**	NeuAca(2-6)Galβ(1-4)GlcNAcβ(1-3)Galβ(1-4)GlcNAcβ(1-3)Galβ(1-4)GlcNAcβ(1-3)Galβ(1-4)GlcNAcβ(1-3)Galβ(1-4)GlcNAcβ(1-3)[NeuAca(2-6)Galβ(1-4)GlcNAcβ(1-3)Galβ(1-4)GlcNAcβ(1-3)Galβ(1-4)GlcNAcβ(1-3)Galβ(1-4)GlcNAcβ(1-3)Galβ(1-4)GlcNAcβ(1-6)]-GalNAca-Thr-NH2	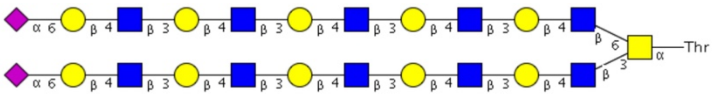
**106**	NeuAca(2-6)Galβ(1-4)GlcNAcβ(1-3)Galβ(1-4)GlcNAcβ(1-3)Galβ(1-4)GlcNAcβ(1-3)Galβ(1-4)GlcNAcβ(1-6)GalNAca-Thr-NH2	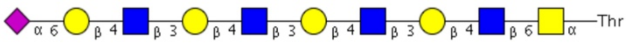
**107**	NeuAca(2-6)Galβ(1-4)GlcNAcβ(1-3)Galβ(1-4)GlcNAcβ(1-3)Galβ(1-4)GlcNAcβ(1-3)Galβ(1-4)GlcNAcβ(1-3)Galβ(1-4)GlcNAcβ(1-6)GalNAca-Thr-NH2	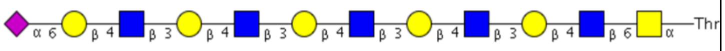
**108**	NeuAca(2-6)Galβ(1-4)GlcNAcβ(1-3)Galβ(1-4)GlcNAcβ(1-3)Galβ(1-4)GlcNAcβ(1-6)[NeuAca(2-6)Galβ(1-4)GlcNAcβ(1-3)Galβ(1-4)GlcNAcβ(1-3)] Galβ(1-4)GlcNAcβ-ethyl-NH2	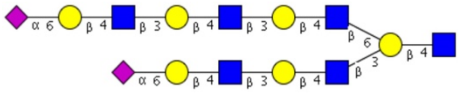
**109**	NeuAca(2-6)Galβ(1-3)GlcNAcβ(1-3)Galβ(1-4)GlcNAcβ(1-6)[NeuAca(2-6)Galβ(1-3)GlcNAcβ(1-3)] Galβ(1-4)GlcNAcβ-ethyl-NH2	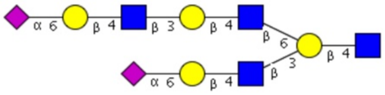
**110**	Galβ(1-4)GlcNAcβ(1-2)Mana(1-3)[NeuAca(2-6)Galβ(1-4)GlcNAcβ(1-2)Mana(1-6)]-Manβ(1-4)GlcNAcβ(1-4)GlcNAcβ-Asn-NH2	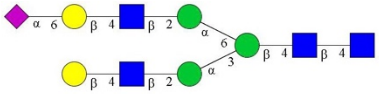
**111**	NeuAca(2-6)Galβ(1-4)GlcNAcβ(1-2)Mana(1-3)[Galβ(1-4)GlcNAcβ(1-2)Mana(1-6)]-Manβ(1-4)GlcNAcβ(1-4)GlcNAcβ-Asn-NH2	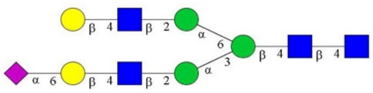
**112**	GlcNAcβ(1-2)Mana(1-3)[NeuAca(2-6)Galβ(1-4)GlcNAcβ(1-2)Mana(1-6)]-Manβ(1-4)GlcNAcβ(1-4)GlcNAcβ-Asn-NH2	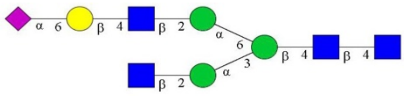
**113**	NeuAca(2-6)Galβ(1-4)GlcNAcβ(1-2)Mana(1-3)[NeuAca(2-6)Galβ(1-4)GlcNAcβ(1-2)Manα(1-6)]-Manβ(1-4)GlcNAcβ(1-4)GlcNAcβ-Asn-NH2	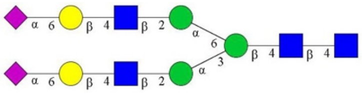
**114**	NeuAca(2-6)Galβ(1-4)GlcNAcβ(1-3)Galβ(1-4)GlcNAcβ(1-2)Mana(1-3)[NeuAca(2-6)Galβ(1-4)GlcNAcβ(1-3)Galβ(1-4)GlcNAcβ(1-2)Mana(1-6)]-Manβ(1-4)GlcNAcβ(1-4)GlcNAcβ-Asn-NH2	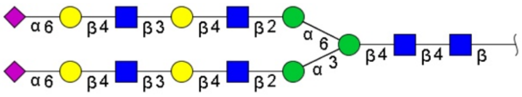
**115**	NeuAca(2-6)Galβ(1-4)GlcNAcβ(1-3)Galβ(1-4)GlcNAcβ(1-2)Mana(1-3)[NeuAca(2-6)Galβ(1-4)GlcNAcβ(1-3)Galβ(1-4)GlcNAcβ(1-2)Mana(1-6)]-Manβ(1-4)GlcNAcβ(1-4)GlcNAcβ-(Lys-Val-Ala)Asn-Lys-Thr-NH2	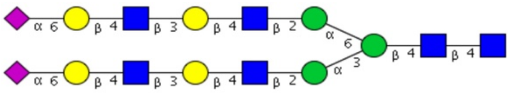

Glycan #	Common Name	Structure
**116**	NeuAca(2-6)Galβ(1-4)GlcNAcβ(1-3)Galβ(1-4)GlcNAcβ(1-3)Galβ(1-4)GlcNAcβ(1-2)Mana(1-3)[NeuAca(2-6)Galβ(1-4)GlcNAcβ(1-3)Galβ(1-4)GlcNAcβ(1-3)Galβ(1-4)GlcNAcβ(1-2)Mana(1-6)]-Manβ(1-4)GlcNAcβ(1-4)GlcNAcβ-Asn-NH2	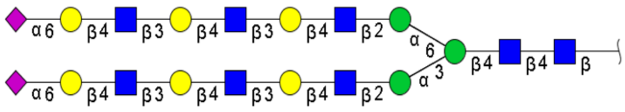
**117**	NeuAca(2-6)Galβ(1-4)GlcNAcβ(1-3)Galβ(1-4)GlcNAcβ(1-3)Galβ(1-4)GlcNAcβ(1-2)Mana(1-3)[NeuAca(2-6)Galβ(1-4)GlcNAcβ(1-3)Galβ(1-4)GlcNAcβ(1-3)Galβ(1-4)GlcNAcβ(1-2)Manα(1-6)]-Manβ(1-4)GlcNAcβ(1-4)GlcNAcβ-(Lys-Val-Ala)Asn-Lys-Thr-NH2	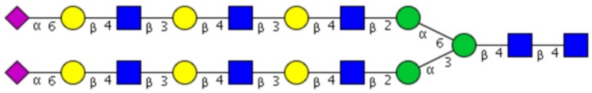
**118**	NeuAca(2-6)Galβ(1-4)GlcNAcβ(1-3)Galβ(1-4)GlcNAcβ(1-3)Galβ(1-4)GlcNAcβ(1-3)Galβ(1-4)GlcNAcβ(1-2)Mana(1-3)[NeuAcα(2-6)Galβ(1-4)GlcNAcβ(1-3)Galβ(1-4)GlcNAcβ(1-3)Galβ(1-4)GlcNAcβ(1-3)Galβ(1-4)GlcNAcβ(1-2)Mana(1-6)]-Manβ(1-4)GlcNAcβ(1-4)GlcNAcβ-(Lys-Val-Ala)Asn-Lys-Thr-NH2	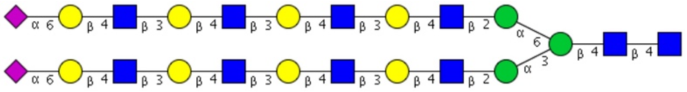
**119**	NeuAca(2-6)Galβ(1-4)GlcNAcβ(1-3)Galβ(1-4)GlcNAcβ(1-3)Galβ(1-4)GlcNAcβ(1-3)Galβ(1-4)GlcNAcβ(1-3)Galβ(1-4)GlcNAcβ(1-2)Mana(1-3)[NeuAca(2-6)Galβ(1-4)GlcNAcβ(1-3)Galβ(1-4)GlcNAcβ(1-3)Galβ(1-4)GlcNAcβ(1-3)Galβ(1-4)GlcNAcβ(1-3)Galβ(1-4)GlcNAcβ(1-2)Mana(1-6)]-Manβ(1-4)GlcNAcβ(1-4)GlcNAcβ-(Lys-Val-Ala)Asn-Lys-Thr-NH2	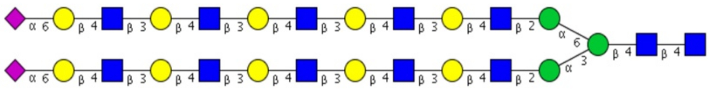
**120**	NeuAca(2-6)Galβ(1-4)GlcNAcβ(1-3)Galβ(1-4)GlcNAcβ(1-2)Mana(1-3)[NeuAca(2-6)Galβ(1-4)GlcNAcβ(1-3)Galβ(1-4)GlcNAcβ(1-2)Mana(1-6)]-Manβ(1-4)GlcNAcβ(1-4)[Fuca(1-6)]-GlcNAcβ-(Lys-Val-Ala)Asn-Lys-Thr-NH2	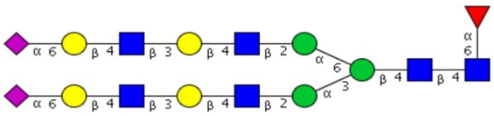
**121**	NeuAca(2-6)Galβ(1-4)GlcNAcβ(1-3)Galβ(1-4)GlcNAcβ(1-3)Galβ(1-4)GlcNAcβ(1-2)Mana(1-3)[NeuAca(2-6)Galβ(1-4)GlcNAcβ(1-3)Galβ(1-4)GlcNAcβ(1-3)Galβ(1-4)GlcNAcβ(1-2)Mana(1-6)]-Manβ(1-4)GlcNAcβ(1-4)[Fuca(1-6)]-GlcNAcβ-(Lys-Val-Ala)Asn-Lys-Thr-NH2	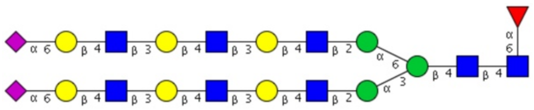

Glycan #	Common Name	Structure
**122**	NeuAcα(2-6)Galβ(1-4)GlcNAcβ(1-3)Galβ(1-4)GlcNAcβ(1-3)Galβ(1-4)GlcNAcβ(1-3)Galβ(1-4)GlcNAcβ(1-2)Manα(1-3)[NeuAcα(2-6)Galβ(1-4)GlcNAcβ(1-3)Galβ(1-4)GlcNAcβ(1-3)Galβ(1-4)GlcNAcβ(1-3)Galβ(1-4)GlcNAcβ(1-2)Manα(1-6)]-Manβ(1-4)GlcNAcβ(1-4)[Fucα(1-6)]-GlcNAcβ-(Lys-Val-Ala)Asn-Lys-Thr-NH2	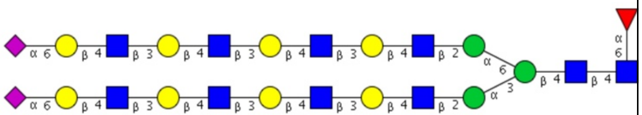
**123**	NeuAcα(2-6)Galβ(1-4)GlcNAcβ(1-3)Galβ(1-4)GlcNAcβ(1-2)Manα(1-3){NeuAcα(2-6)Galβ(1-4)GlcNAcβ(1-3)Galβ(1-4)GlcNAcβ(1-2)[NeuAcα(2-6)Galβ(1-4)GlcNAcβ(1-3)Galβ(1-4)GlcNAcβ(1-6)Manα(1-6)]}-Manβ(1-4)GlcNAcβ(1-4)GlcNAcβ-(Lys-Val-Ala)Asn-Lys-Thr-NH2	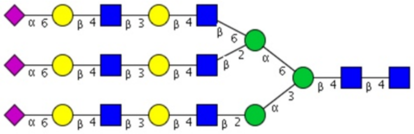
**124**	NeuAcα(2-6)Galβ(1-4)GlcNAcβ(1-3)Galβ(1-4)GlcNAcβ(1-2)Manα(1-3){NeuAcα(2-6)Galβ(1-4)GlcNAcβ(1-3)Galβ(1-4)GlcNAcβ(1-2)[NeuAcα(2-6)Galβ(1-4)GlcNAcβ(1-3)Galβ(1-4)GlcNAcβ(1-6)Manα(1-6)]}-Manβ(1-4)GlcNAcβ(1-4)[Fucα(1-6)]-GlcNAcβ-(Lys-Val-Ala)Asn-Lys-Thr-NH2	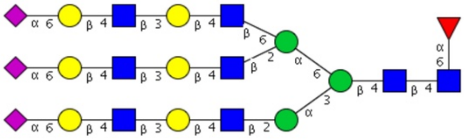
**125**	NeuAcα(2-6)Galβ(1-4)GlcNAcβ(1-3)Galβ(1-4)GlcNAcβ(1-3)Galβ(1-4)GlcNAcβ(1-2)Manα(1-3){NeuAcα(2-6)Galβ(1-4)GlcNAcβ(1-3)Galβ(1-4)GlcNAcβ(1-3)Galβ(1-4)GlcNAcβ(1-2)[NeuAcα(2-6)Galβ(1-4)GlcNAcβ(1-3)Galβ(1-4)GlcNAcβ(1-3)Galβ(1-4)GlcNAcβ(1-6)Manα(1-6)]}-Manβ(1-4)GlcNAcβ(1-4)[Fucα(1-6)]-GlcNAcβ-(Lys-Val-Ala)Asn-Lys-Thr-NH2	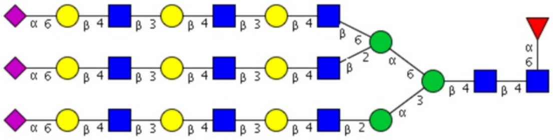
**126**	LN/6'SLN/6'SLN-TriN	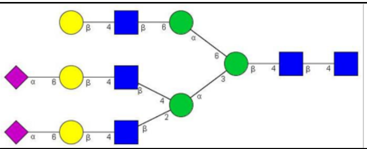
**127**	6'SLN/LeX/LeX-TriN	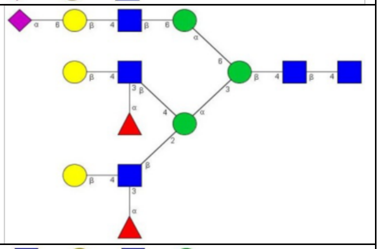
**128**	6'SLNLN/LeX/LeX-TriN	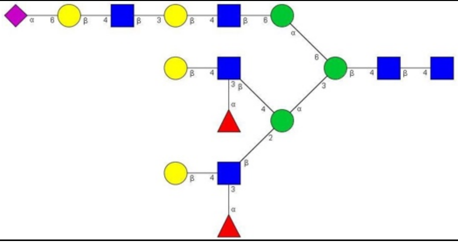
